# Biostimulant Modulation of Soil Mycobiome Structure and Diversity and *Amaranthus cruentus* Growth Under Perfluorotetradecanoic Acid Stress

**DOI:** 10.3390/molecules31193385

**Published:** 2026-09-23

**Authors:** Małgorzata Baćmaga, Jadwiga Wyszkowska, Edyta Boros-Lajszner, Jan Kucharski

**Affiliations:** Department of Soil Science and Microbiology, Faculty of Agriculture and Forestry, University of Warmia and Mazury in Olsztyn, Plac Łódzki 3, 10-727 Olsztyn, Poland; m.bacmaga@uwm.edu.pl (M.B.); edyta.boros@uwm.edu.pl (E.B.-L.); jan.kucharski@uwm.edu.pl (J.K.)

**Keywords:** soil fungi, perfluorotetradecanoic acid, soil environmental contamination, fungal taxonomic diversity, ecosystem functions of fungi, plant

## Abstract

Perfluorotetradecanoic acid (PA) is a long-chain perfluorocarboxylic acid belonging to the group of per- and polyfluoroalkyl substances (PFASs), whose exceptional chemical stability promotes their persistence and accumulation in the environment. Despite the growing interest in PFASs, knowledge of the impact of PA on soil fungal communities and crop plants remains limited. The aim of this study was to evaluate the effects of PA and the biostimulants Shigeki and Aminoprim on the structure, diversity, and functional potential of the soil mycobiome, as well as on the growth and development of *Amaranthus cruentus*. A pot experiment was conducted under controlled greenhouse conditions using Eutric Cambisols soil. The structure of fungal communities was determined using high-throughput sequencing of the ITS region, while diversity was assessed based on alpha and beta diversity indices and trophic group analyses. In plants, stem length and the biomass of aboveground parts and roots were analyzed. PA significantly altered the composition and structure of soil fungal communities. Fungi belonging to the phylum *Ascomycota* were dominant in all samples, with their highest abundance observed in PA-contaminated soil. Contamination with this compound also reduced the abundance of the genera *Penicillium* and *Talaromyces*, while increasing the relative abundance of *Humicola*, *Chaetomium*, and *Fusarium* in soil treated with PA and biostimulants. Aminoprim promoted an increase in taxonomic richness and community evenness, whereas Shigeki enhanced the abundance of *Chytridiomycota* in PA-contaminated soil. Perfluorotetradecanoic acid inhibited the growth of *A. cruentus*; however, the applied biostimulants partially mitigated its adverse effects. The results indicate that PA affects both the structure and functioning of the soil mycobiome as well as plant development, while biostimulation may represent a potential strategy for supporting the biological stability of soils subjected to PFAS contamination.

## 1. Introduction

Perfluoroalkyl substances (PFASs) are organic compounds that, due to their high chemical stability, resistance to degradation, and bioaccumulative potential, are considered one of the most serious contemporary environmental threats [1,2]. Particular attention has been focused on long-chain perfluorocarboxylic acids, which are widely detected in water, sediments, and soils [2]. Compared with their short-chain analogues, these compounds exhibit greater hydrophobicity, a higher potential for bioaccumulation, and stronger affinity for organic matter. This group includes perfluorotetradecanoic acid (PA), whose high persistence results from the presence of stable carbon–fluorine bonds and its strong adsorption onto soil particles, which may lead to long-term impacts on soil organisms [3]. PFASs accumulate in soil, one of the most important environmental compartments responsible for their retention, and this accumulation may affect both the physicochemical properties of soil and the functioning of the soil microbiome. An increasing number of studies indicate that PFAS exposure leads to changes in the taxonomic composition of microbial communities, reductions in species richness, and disturbances in interactions among microorganisms [4,5,6]. A decline in biodiversity indices and the selection of microorganisms with greater tolerance to these compounds have also been demonstrated, resulting in simplified community structures and reduced functional diversity. Furthermore, PFASs may influence the structure of ecological networks formed by soil microorganisms by decreasing their complexity and stability, thereby limiting ecosystem resilience to environmental stressors [7,8,9]. The effects of these compounds on microorganisms may occur through both direct and indirect mechanisms. Direct mechanisms include disruption of cell membrane integrity, induction of oxidative stress, and disturbances in cellular homeostasis, whereas indirect effects result from changes in soil physicochemical properties, such as enzymatic activity, nutrient availability, and soil aggregate structure [1,2].

A particularly important component of the soil microbiome is fungi, which play a crucial role in maintaining the proper functioning of terrestrial ecosystems. They contribute to the decomposition of organic matter, nutrient mineralization, carbon cycling, and the formation of soil structure through the development of extensive hyphal networks. Mycorrhizal fungi also perform essential functions by establishing close symbiotic relationships with plants, thereby enhancing mineral nutrient uptake efficiency and increasing host resistance to adverse environmental conditions [2,10].

Exposure of fungi to PFASs may induce changes in fungal growth rates, metabolic activity, and population structure, manifested as reduced taxonomic diversity and shifts in the dominance of specific functional groups. Such disturbances may disrupt biogeochemical processes and reduce the functional stability of soil ecosystems [10]. At the same time, soil fungi demonstrate the ability to develop adaptive mechanisms in response to the presence of persistent chemical contaminants in the soil. These microorganisms possess a complex set of strategies that enable their survival and functioning in PFAS-contaminated environments [11]. Despite the growing number of studies investigating the occurrence of PFASs and related processes, the impact of PA on the structure, diversity, and metabolic functions of soil fungi remains poorly understood [2,10].

Perfluorotetradecanoic acid (PA) was selected as the subject of this study due to its specific environmental properties associated with the length of its perfluoroalkyl chain, high persistence, and potential for bioaccumulation, while its environmental fate and ecological significance remain insufficiently understood. Previous studies have focused primarily on perfluorooctanoic acid (PFOA) and perfluorooctanesulfonate (PFOS), for which the most extensive information is available regarding their occurrence, transport, bioaccumulation, and effects on organisms. In recent years, however, increasing attention has been devoted to long-chain PFAS homologues, including perfluorotetradecanoic acid (PA), whose environmental properties and potential ecological significance remain poorly understood. Differences in perfluoroalkyl chain length and functional group type influence sorption properties, environmental mobility, bioaccumulation potential, and interactions with organisms, highlighting the need to expand knowledge of less-studied long-chain PFASs as well [12]. PFOA and PFOS each contain eight carbon atoms in their perfluoroalkyl chains, whereas PA has a substantially longer 14-carbon chain. As perfluoroalkyl chain length increases, hydrophobicity and affinity for organic matter and the solid phase generally increase, which may promote retention in soil and sediments while limiting mobility in the aqueous phase. Consequently, PFOA is generally more mobile and more readily transported in aquatic environments, whereas PA may exhibit a stronger affinity for the solid phase and may persist in soil and sediments for extended periods [13,14]. These differences may also be reflected in their bioaccumulation potential. As a long-chain PFAS, PA may have a greater potential for accumulation in organisms than PFOA. For example, studies conducted on fish (*Cyprinus carpio* L.) reported a bioconcentration factor for PA ranging from 16,000 to 17,000 dm^3^ kg^−1^, compared with 10,000–16,000 dm^3^ kg^−1^ for PFOA [15]. In the case of PA, strong association with the solid phase may limit its bioavailability while simultaneously promoting long-term retention in the soil environment and potentially facilitating trophic transfer. Thus, PFOA is generally characterized by greater environmental mobility, whereas PFOS has a high bioaccumulation potential and PA exhibits strong affinity for the solid phase and potential for bioaccumulation, making it an environmentally relevant compound for ecological risk assessment [16]. In recent years, increasing attention has been given to the use of biostimulants as tools supporting the restoration of biological activity in soils exposed to various stress factors. These preparations may stimulate microbial development, improve nutrient availability, and enhance the resistance of soil organisms to adverse environmental conditions [17,18,19]. In the present study, the biostimulants Shigeki and Aminoprim were used. Shigeki is a plant biostimulant intended for use under stress conditions. It contains a concentrated extract of the marine alga *Ascophyllum nodosum* along with micronutrients, including phosphorus, potassium, boron, molybdenum, copper, iron, manganese, and zinc. *A. nodosum* extract can act as a biostimulant, affecting both plant growth and development and the functioning of the soil environment. The bioactive compounds it contains, including polysaccharides, polyphenols, osmolytes, and substances with growth regulator-like activity, may stimulate root system development, improve the efficiency of mineral nutrient and water uptake, and enhance plant tolerance to abiotic stress. Particular importance has been attributed to the effects of the extract on the rhizosphere, as its application may modify the composition and activity of microorganisms associated with plant roots. *A. nodosum* extract may promote the development of beneficial microorganisms and alter the composition of root exudates, potentially strengthening plant–microbiome interactions [20,21]. In addition, polysaccharides present in the extract, particularly alginates, may positively affect soil water retention and structure, thereby improving conditions for root development. The application of the extract may also enhance nutrient-use efficiency, including nitrogen-use efficiency, creating the potential to reduce mineral fertilizer inputs without substantially decreasing plant productivity. Consequently, the use of *A. nodosum* as a biostimulant may contribute to reducing nutrient losses and mitigating the environmental pressure associated with intensive fertilization [22]. In contrast, Aminoprim contains amino acids belonging to a group of organic compounds that are essential for proper plant functioning. They not only serve as basic components of proteins but also participate in numerous metabolic and regulatory processes. Individual amino acids may perform different, often multifunctional, roles in plants. They are involved, among other processes, in water regulation, the stimulation of growth and germination, the synthesis of chlorophyll, plant hormones, polyamines, and lignin, as well as mechanisms involved in responses to abiotic and biotic stress. Proline and hydroxyproline are of particular importance in mitigating the effects of unfavorable environmental conditions, as they participate in water regulation and the stabilization of cellular structures. Glutamate, in turn, may act as a growth stimulant and as a source of organic nitrogen used in the synthesis of other amino acids and proteins. Some amino acids, such as glycine, serine, alanine, and lysine, participate in chlorophyll metabolism, whereas phenylalanine is a precursor of lignin biosynthesis. Amino acids may also act as precursors of growth regulators, including auxins, polyamines, and ethylene, thereby influencing plant growth, generative development, and reproductive processes [23]. Amino acids may also affect the functioning of the soil environment by modifying microbial activity, nutrient availability, and processes occurring in the rhizosphere. They provide readily available sources of nitrogen and carbon for plants and microorganisms, supporting mineralization and nitrogen transformation processes. Their presence may also increase the activity of microorganisms involved in organic matter decomposition and nutrient cycling. Furthermore, amino acids may influence soil physicochemical properties through the binding of mineral ions and by affecting their availability to plants. In the rhizosphere, they may shape the composition and activity of microbial communities, potentially promoting more efficient nutrient utilization and improving conditions for plant growth. However, these effects depend on the type and dose of amino acids and on soil properties [24]. Previous studies on the effects of PFASs on the soil environment have focused primarily on their fate and accumulation, as well as their potential effects on plants and selected groups of microorganisms, whereas knowledge regarding the effects of PA on the structure of soil fungal communities remains limited. In particular, the relationships between the presence of PA in soil, plant responses, and changes in the soil mycobiome, as well as the potential for these relationships to be modified by the application of biostimulants, remain poorly understood. In this context, the novelty of the present study lies in the integrated assessment of the responses of *Amaranthus cruentus* and fungal communities to the presence of perfluorotetradecanoic acid in soil, while simultaneously considering the effects of biostimulants. This approach enables the relationships between contaminant-induced stress, plant responses, and changes in the soil mycobiome to be examined, providing new insights into the ecological consequences of perfluorotetradecanoic acid in the soil environment.

It was hypothesized that soil contamination with perfluorotetradecanoic acid (PA) would induce significant changes in the structure and diversity of fungal communities and adversely affect the growth and development of *Amaranthus cruentus*. At the same time, it was hypothesized that the application of the biostimulants Shigeki and Aminoprim could mitigate the adverse effects of PA-induced stress by modifying the structure and diversity of the soil mycobiome and improving the growth and physiological condition of *A. cruentus*.

This study aimed to evaluate the potential of the biostimulants Shigeki (Sh) and Aminoprim (Am) to mitigate the adverse effects of perfluorotetradecanoic acid (PA) on the soil mycobiome and *Amaranthus cruentus*, with particular emphasis on changes in the structure and diversity of fungal communities and plant growth and physiological condition.

## 2. Results

### 2.1. Structure of Fungal Communities in Soil Subjected to Perfluorotetradecanoic Acid and Biostimulant Treatments

In all analyzed samples, fungi belonging to the phylum *Ascomycota* were dominant; however, their relative abundance varied depending on the experimental conditions (Figure 1). In the C treatment (soil without the addition of perfluorotetradecanoic acid or biostimulants), *Ascomycota* accounted for 88.85% of the fungal community, and their relative abundance increased further in the PA and C_Sh treatments. In contrast, a pronounced decrease was observed in the PA_Sh treatment, where *Ascomycota* represented only 29.43% of the fungal community. In the PA_Am treatment, their relative abundance remained relatively high (78.83%). The most pronounced changes were observed for fungi belonging to the phylum *Chytridiomycota*. Their abundance was negligible in the C treatment, whereas they became highly represented in the PA_Sh treatment, accounting for 59.79% of the fungal community. In the remaining treatments, *Chytridiomycota* occurred at low relative abundances, indicating a strong response to the combined effects of PA contamination and the Shigeki biostimulant. *Basidiomycota* occurred at relatively low abundance across all treatments, with the highest proportion observed in PA_Am (5.59%). *Mortierellomycota* and *Rozellomycota* were also detected at low relative abundances, although their proportions increased in the PA_Am treatment. The group designated as “*Others*” also varied among treatments, reaching its highest proportion in PA_Am (9.90%). Overall, the results indicate that both PA contamination and biostimulant application substantially modified fungal community structure at the phylum level. The most pronounced changes involved the relative abundances of *Ascomycota* and, particularly, *Chytridiomycota*, with the latter showing a strong response to the combined application of PA and the Shigeki biostimulant.

Principal component analysis (PCA) was performed to evaluate changes in the structure of fungal communities at the phylum level in response to perfluorotetradecanoic acid and the biostimulants applied (Figure 2). The first two principal components explained 97.84% of the total variance, with PCA1 accounting for 69.83% and PCA2 for 28.01%. PCA1 represented the main axis of sample differentiation and showed a strong negative correlation with the variables C, PA, C_Sh, and PA_Am, whereas PCA2 was most strongly associated with the PA_Sh treatment and the *Rozellomycota* phylum. The distribution of fungal phyla indicated diverse responses to the experimental treatments analyzed. *Ascomycota* showed the strongest association with the C, PA, C_Sh, and PA_Am treatments. *Basidiomycota* and the group designated as “*Others*” were also related to these variants, although to a lesser extent. *Chytridiomycota* was positioned opposite along the PCA1 axis and exhibited a closer relationship with the PA_Sh and C_Am treatments. In contrast, *Mortierellomycota* and *Rozellomycota* showed weaker associations with the C, PA, and C_Sh treatments, indicating a distinct response pattern of these fungal phyla compared with the remaining groups.

The analysis of taxonomic structure at the genus level revealed significant differences in fungal community composition among the studied treatments (Figure 3). In all samples, fungi belonging to the genera *Penicillium* and *Talaromyces* were dominant; however, their relative abundances varied considerably depending on the experimental treatment. *Penicillium* was most abundant in the C and C_Sh treatments, accounting for approximately 45.95–49.19% of the fungal community. In contrast, its abundance decreased substantially in the PA and PA_Am treatments, where it accounted for 20.49% and 22.26%, respectively. A similar pattern was observed for *Talaromyces*, which was most abundant in the C and C_Sh treatments, whereas its relative abundance was considerably lower in the remaining treatments. In the PA_Sh and PA_Am treatments, a pronounced increase in the relative abundances of *Humicola*, *Chaetomium*, and *Fusarium* was observed. The strongest response was observed for *Humicola*, which reached 11.27–12.21% in these treatments, while remaining below 1.00% in the other soil samples. *Chaetomium* also increased markedly, particularly in PA_Sh, where its relative abundance reached 8.90%. Similarly, *Fusarium* showed higher relative abundances in the PA_Sh and PA_Am treatments, reaching 9.10% and 10.36%, respectively, compared with considerably lower levels in the C and PA treatments. Other fungal genera, including *Trichoderma*, *Exophiala*, *Oidiodendron*, *Acremonium*, *Setophoma*, and *Tetragoniomyces*, occurred at relatively low abundances. An important component of the fungal communities was the group designated as “*Others*”, which increased substantially in the PA_Sh and PA_Am treatments, accounting for more than 30.00% of the community in both cases. This increase may indicate greater taxonomic heterogeneity and community complexity under these experimental conditions. Overall, the experimental treatments significantly influenced fungal community structure at the genus level. PA contamination combined with the Shigeki or Aminoprim biostimulants resulted in a shift away from the dominance of *Penicillium* and *Talaromyces* toward a greater contribution of saprotrophic and potentially cellulolytic taxa, particularly *Humicola*, *Chaetomium*, and *Fusarium*.

The co-occurrence network analysis revealed numerous associations among the analyzed fungal genera (Figure 4). Most genera were connected to multiple taxa, indicating a complex structure of the analyzed co-occurrence network. Node size reflected differences in the number of connections of individual taxa within the network. The largest nodes represented the genera *Solicoccozyma*, *Tetragoniomyces*, *Exophiala*, and *Acremonium*, which were characterized by numerous connections with other genera. These taxa occupied prominent positions within the analyzed network. The genera *Oidiodendron*, *Parachaetomium*, *Penicillium*, and *Trichoderma* were also located in the central part of the network and exhibited numerous connections with other nodes. Their positions in the network indicate frequent co-occurrence with other analyzed taxa. Although the genera *Chaetomium* and *Humicola* were positioned more peripherally, they remained connected to numerous network elements, indicating their contribution to the observed co-occurrence structure. The smallest nodes represented the genera *Albifimbria*, *Fusarium*, *Setophoma*, and *Talaromyces*. These taxa had fewer connections than the most highly connected nodes but still exhibited associations with other fungal genera. Overall, the network revealed a complex pattern of co-occurrence among the analyzed genera, with *Solicoccozyma*, *Tetragoniomyces*, *Exophiala*, and *Acremonium* standing out in terms of the number of connections and their positions within the network structure.

### 2.2. Alpha Diversity of Fungi in Soil Subjected to Perfluorotetradecanoic Acid and Biostimulant Treatments

Analysis of alpha diversity indices showed that perfluorotetradecanoic acid influenced the diversity and structure of soil fungal communities, and the magnitude of this effect depended on the biostimulant applied (Figure 5). The indices used enabled the assessment of both the number of taxa present in the samples and the degree of evenness in their distribution, as well as the dominance of particular fungal groups.

The Shannon index values ranged from 3.327 to 5.434. The application of the tested acid without the addition of a biostimulant resulted in a decrease in this index to 3.327 in the PA treatment, indicating a reduction in fungal alpha diversity. The introduction of the Aminoprim biostimulant significantly increased the Shannon index value to 5.434 in the PA_Am treatment, demonstrating an increase in both taxonomic richness and distributional evenness among taxa. In contrast, the application of the Shigeki biostimulant did not produce a comparable effect. A similar trend was observed in the perfluorotetradecanoic acid treatments, where the Shannon index increased from 4.327 in the C treatment to 4.736 after the application of Aminoprim (C_Am), whereas after the application of Shigeki, the value reached 4.057 (C_Sh).

A similar pattern was observed for the Simpson index, with values ranging from 0.683 to 0.950. The highest value was recorded in the PA_Am treatment (0.950), indicating the highest community diversity and the lowest dominance of individual taxa. In the C soil sample, the index value was 0.889 and increased to 0.918 after the application of the Aminoprim biostimulant (C_Am). In contrast, the application of the Shigeki biostimulant in perfluorotetradecanoic acid-contaminated soil (PA_Sh) resulted in a decrease in the index value to 0.683, indicating a greater dominance of a limited number of taxa. The obtained results suggest that the Aminoprim biostimulant was more effective than Shigeki in mitigating changes in fungal community structure induced by the tested acid, thereby promoting the maintenance of higher biological diversity.

A similar trend was observed for Pielou’s index. In soil containing perfluorotetradecanoic acid, the index value was 0.422 (PA), whereas the application of Aminoprim in the acid-treated soil (PA_Am) increased it to 0.637, indicating a more balanced distribution of individual taxa. Following the application of Aminoprim in the C_Am treatment, the index value increased slightly from 0.578 to 0.582, whereas the addition of Shigeki resulted in a decrease to 0.414 in the PA_Sh treatment. These results indicate that Aminoprim was more effective in alleviating disturbances in fungal community structure caused by perfluorotetradecanoic acid contamination.

The analysis of taxonomic richness indices showed that the number of observed features (Observed features) ranged from 180.00 to 370.00, while Chao1 index values ranged from 180.00 to 375.16. In soil contaminated with perfluorotetradecanoic acid (PA), the application of the Aminoprim biostimulant (PA_Am) increased the number of observed features to 370.00 and the Chao1 index value to 375.16. In the C treatment, the number of observed features increased from 180.00 to 283.00 following the application of Aminoprim (C_Am), while the Chao1 index increased from 180.00 to 301.529. The Shigeki biostimulant also increased taxonomic richness; however, this effect was less pronounced, as the PA_Sh treatment exhibited 317.00 observed features and a Chao1 index value of 342.021. The dominance index ranged from 0.050 to 0.317. The lowest value was recorded after the application of Aminoprim in perfluorotetradecanoic acid-contaminated soil (0.050), indicating a reduction in the dominance of individual taxa and a more balanced community structure. In the C treatment, the application of Aminoprim reduced the dominance index from 0.111 to 0.082. In contrast, the highest values were observed in the PA_Sh (0.317) and PA (0.258) treatments, indicating a stronger dominance of a limited number of taxa.

High Good’s coverage values (0.998–1.000) obtained for all analyzed treatments confirm the high completeness of sequencing and sufficient sampling depth, ensuring a reliable assessment of fungal community diversity.

The obtained results indicate that the tested acid reduced fungal community diversity and evenness while increasing the dominance of selected taxa, whereas the application of the Aminoprim biostimulant effectively mitigated these adverse changes. The effect of the Shigeki biostimulant was weaker and did not lead to improvements in diversity parameters across all analyzed indices.

### 2.3. Beta Diversity of Fungi in Soil Subjected to Perfluorotetradecanoic Acid and Biostimulant Treatments

Analysis of beta diversity revealed significant differences in the structure and composition of soil fungal communities among the treatments analyzed (Figure 6, Figure 7, Figure 8 and Figure 9). The application of four complementary distance measures (Bray–Curtis, Jaccard, unweighted UniFrac, and weighted UniFrac) enabled a comprehensive assessment of differences in taxonomic composition and phylogenetic relationships, as well as the relative abundance of individual taxa.

Principal Coordinates Analysis (PCoA) based on Bray–Curtis distances showed differences in fungal community composition among the analyzed treatments (Figure 6). The first two principal coordinates explained 48.38% of the total variation, with 29.17% accounted for by PCo1 and 19.21% by PCo2. Samples C, C_Sh, and C_Am clustered closely together, indicating relatively similar fungal community compositions among these treatments. In contrast, samples PA and PA_Sh formed a distinct cluster. PA_Am was clearly separated from the other treatments, particularly along the PCo1 axis, suggesting a distinct fungal community structure (Figure 6a). The Bray–Curtis distance matrix supported the pattern observed in the PCoA (Figure 6b). Pairwise distances between samples ranged from 0.642 to 0.723. The smallest distance was observed between C_Am and C_Sh (0.642), indicating the greatest similarity in fungal community composition between these treatments, whereas the largest distance was recorded between C and PA_Am (0.723).

Analysis of fungal community composition based on Jaccard distances showed clear differences among the analyzed treatments (Figure 7). The first two ordination axes accounted for 49.31% of the total variation, with 29.85% explained by PCo1 and 19.46% by PCo2. The ordination plot showed a clear separation of treatments C, PA, and C_Sh from C_Am and PA_Am, which were located in a distinct region of the ordination space (Figure 7a). The PA_Sh treatment was particularly distinct from the other treatments, being positioned at the extreme end of the ordination plot. Jaccard distance values indicated relatively high dissimilarity among the analyzed treatments (Figure 7b). The smallest distance was observed between C and C_Sh (0.653), whereas the largest was recorded between PA and PA_Sh (0.792). The high distance values between PA_Sh and the other treatments (0.704–0.792) were consistent with its clear separation in the ordination space. Overall, PA_Sh exhibited the most distinct fungal community composition, whereas C, PA, and C_Sh showed greater similarity in terms of fungal community composition.

Beta diversity analysis based on the Unweighted UniFrac metric revealed differences in the phylogenetic structure of fungal communities among the analyzed treatments (Figure 8). The first two PCoA axes explained 57.86% of the total variation, with 38.67% explained by PCo1 and 19.19% by PCo2. Samples C_Sh and PA formed a compact cluster, as did C_Am and PA_Am, whereas C and PA_Sh were clearly separated from the other treatments (Figure 8a). The PCoA results were consistent with the Unweighted UniFrac distance matrix. The smallest distances were observed between C_Am and PA_Am (0.476), C_Sh and PA (0.481), and C and C_Sh (0.497), whereas the largest distances were recorded between C and PA_Sh (0.647), PA and PA_Sh (0.607), and C and C_Am (0.605). Thus, PA_Sh exhibited the greatest phylogenetic dissimilarity from the other treatments, whereas C_Am and PA_Am showed the greatest similarity. Overall, the observed patterns indicate differences in the phylogenetic structure of fungal communities among the experimental treatments (Figure 8b).

Analysis based on the Weighted UniFrac metric revealed differences among the analyzed treatments in both the phylogenetic structure of fungal communities and the relative abundance of fungal taxa (Figure 9). The first two PCoA axes explained 86.28% of the total variation, with 68.12% explained by PCo1 and 18.16% by PCo2, indicating that these two axes captured a large proportion of the variation among the analyzed communities. Samples C and C_Sh formed the most similar group, whereas PA and PA_Am formed a distinct cluster. C_Am exhibited a partially distinct community structure, while PA_Sh was clearly separated from the other treatments (Figure 9a). The Weighted UniFrac distance matrix supported the patterns observed in the PCoA. The smallest distance was observed between C and C_Sh (0.093), whereas the largest distances were recorded between PA_Sh and PA (0.825), C_Sh (0.792), C (0.785), and PA_Am (0.784). Thus, PA_Sh exhibited the greatest dissimilarity in terms of both phylogenetic community structure and the relative abundance of fungal taxa, whereas C and C_Sh showed the greatest similarity (Figure 9b).

### 2.4. Trophic Characteristics of Fungi in Soil Subjected to Perfluorotetradecanoic Acid and Biostimulant Treatments

Analysis of predicted trophic functions showed that saprotrophic fungi dominated across all soil samples (Figure 10, Table S1). The most abundant functional group was Undefined Saprotroph, with the highest relative abundance observed in the PA treatment, where it accounted for 75.21% of the fungal community. Relatively high proportions were also recorded in the C and C_Sh treatments, whereas a pronounced decrease was observed in PA_Sh. A considerable proportion of the fungal community comprised taxa that could not be assigned to a specific trophic function (Unassigned). This group was particularly prominent in the PA_Sh treatment, where it accounted for 80.34% of the community. Its relative abundance was also high in the C_Am and PA_Am treatments, reaching approximately 46–49%. In the remaining treatments, the proportion of unassigned taxa was considerably lower. Among the other functional groups, multifunctional fungi classified as Plant Pathogen–Soil Saprotroph–Wood Saprotroph were relatively abundant, particularly in the PA_Am treatment, where they accounted for 8.94% of the community. The Undefined Saprotroph–Wood Saprotroph group also increased markedly in PA_Am, reaching 10.54%, while remaining at low levels in the other treatments. Mycorrhizal fungi occurred at low relative abundance in all analyzed soil samples. Ericoid and ectomycorrhizal fungi were detected only at low proportions, whereas arbuscular mycorrhizal fungi were virtually absent. Overall, the results indicate differences in the trophic structure of fungal communities among the experimental treatments, particularly in the relative proportions of undefined and unassigned taxa. The results indicate a clear dominance of saprotrophic fungi in the analyzed soil samples, with a relatively minor contribution of mycorrhizal and pathogenic fungi. At the same time, the PA_Sh treatment was characterized by a very high proportion of taxa unassigned to specific trophic functions, whereas the PA_Am treatment exhibited an increased contribution of multifunctional groups associated with organic matter decomposition.

### 2.5. Response of Amaranthus cruentus to Perfluorotetradecanoic Acid and Biostimulant Treatments

The results showed that PA and the applied biostimulants significantly affected the growth and fresh biomass of the aboveground parts and roots of *A. cruentus* (Table 1). The greatest stem length was recorded in the C treatment, where it reached 71.12 cm. The application of PA to the soil (PA treatment) resulted in a significant inhibition of plant growth, leading to a reduction in stem length to 55.11 cm. Stem lengths in the C_Sh, PA_Sh, C_Am, and PA_Am treatments, where stem lengths ranged from 56.46 to 59.92 cm.

The fresh biomass of the aboveground plant parts differed significantly among the analyzed experimental treatments. The highest value was recorded in C_Sh (80.65 g pot^−1^), indicating a beneficial effect of the Shigeki biostimulant on aboveground biomass production. In the PA and C_Am treatments, the biomass of aboveground parts was 72.18 and 72.19 g pot^−1^, respectively. Slightly lower values were recorded in the C treatment (68.95 g pot^−1^) and in the PA_Am treatment (66.18 g pot^−1^). The lowest value of this parameter was obtained in the PA_Sh treatment (60.81 g pot^−1^), which may indicate an adverse effect of the combined application of PA and the Shigeki biostimulant on the development of aboveground plant biomass (Table 1).

A similar trend was observed for the fresh root biomass of the plants (Table 2). The highest root biomass was obtained in the C_Sh treatment (25.81 g pot^−1^), which was significantly higher than that recorded in the other treatments. In the C and PA treatments, fresh root biomass amounted to 22.42 and 21.33 g pot^−1^, respectively, with no significant differences observed between these treatments. The lowest values were recorded in the PA_Sh, C_Am, and PA_Am treatments, where root biomass reached 19.38 g pot^−1^, 20.88 g pot^−1^ and 20.96 g pot^−1^, respectively.

The leaf greenness index (SPAD) values in *Amaranthus cruentus* varied depending on the experimental treatment (Figure 11). In the control treatment (C) and following the application of perfluorotetradecanoic acid (PA), SPAD values remained at similar levels, indicating no significant effect of this compound on the analyzed parameter. Similarly, the application of the Shigeki biostimulant to control soil (C_Sh) did not result in significant changes in SPAD values compared with the control treatment (C). The lowest SPAD value was recorded in the PA_Sh treatment, in which PA and the Shigeki biostimulant were applied simultaneously. A different trend was observed in the treatments involving the Aminoprim biostimulant. Both C_Am and PA_Am exhibited higher SPAD values than corresponding treatments without Aminoprim, indicating a beneficial effect of the Aminoprim biostimulant on this physiological parameter of the plants.

Correlation matrix analysis revealed significant relationships among the analyzed parameters (Figure 12). The strongest positive correlations were observed between Fus and Hum (r = 0.98) and between Bas and Roz (r = 0.97). Other strong positive correlations were found between Tri and Sol (r = 0.94), as well as between Mor and Fus, Mor and Hum, and Acr and SL (r = 0.91). Positive correlations were also observed between Acr and Tet and between Tet and SL (r = 0.90), between Cha and Oid and between FBAP and FRB (r = 0.88), between Tal and FRB (r = 0.87), and between Pen and FRB and Par and Oid (r = 0.83). Strong negative correlations were observed between Asc and Chyt (r = −0.97), Asc and Sol (r = −0.95), Asc and Tri (r = −0.91), and Asc and Exo (r = −0.87). Among the analyzed parameters, the strongest positive correlation involving a plant parameter was observed between FBAP and FRB (r = 0.88). The SPAD parameter was not significantly correlated with any of the other analyzed parameters.

## 3. Discussion

### 3.1. Structure of Fungal Communities in Soil Subjected to Perfluorotetradecanoic Acid and Biostimulant Treatments

The results demonstrated that perfluorotetradecanoic acid contamination and the application of biostimulants significantly affected the structure of soil fungal communities at both the phylum and genus levels, leading to substantial taxonomic shifts and changes in the functional potential of the mycobiome. The observed dominance of the phylum *Ascomycota* across all soil samples is consistent with current scientific knowledge, according to which members of this taxon are among the most resilient and ecologically versatile components of the soil microbiome, particularly under conditions of chemical stress caused by the accumulation of persistent organic pollutants. The dominance of the phylum *Ascomycota* across all soil samples is consistent with current scientific knowledge. Members of this taxon are among the most resilient and ecologically versatile components of the soil microbiome, particularly under chemical stress caused by persistent organic pollutants [25]. A significant finding of the present study was the pronounced response of fungi belonging to the phylum *Chytridiomycota* to the combined effects of perfluorotetradecanoic acid and Shigeki. Their abundance increased markedly in the PA_Sh treatment. In PFAS-contaminated environments, these fungi may serve as sensitive bioindicators of ecosystem disturbance and dynamic changes in the availability of readily assimilable organic matter. Taxa within *Chytridiomycota* are highly sensitive to environmental fluctuations but can also respond rapidly to chemical stress. This may explain the increased abundance of this group under the combined influence of contamination and biostimulation [26]. Such a pronounced increase in the relative abundance of *Chytridiomycota* in the PA_Sh treatment may be associated with several ecological factors. *Chytridiomycota* include, among others, saprotrophic species that utilize organic matter, and their occurrence may depend on substrate availability, nutrient availability, and soil moisture. Water availability may be particularly important because it enables the movement of motile zoospores and colonization of substrates. The availability of nitrogen and phosphorus may additionally influence their growth and zoospore production [27]. The increase in the relative abundance of *Chytridiomycota* from 0.05% to 59.79% represents a change in their proportion within the fungal community and does not necessarily indicate an actual increase in their biomass. Such a substantial change may have partly resulted from a reduction in the relative abundance of other fungal groups under PA exposure, consequently increasing the relative contribution of *Chytridiomycota* to the overall fungal community. At the same time, the observed increase in *Chytridiomycota* in the PA_Sh treatment suggests that the combination of PA and the Shigeki preparation may have been associated with an increase in the relative abundance of this fungal group. Therefore, the observed increase in the relative abundance of *Chytridiomycota* may reflect their response to the altered environmental conditions resulting from PA exposure and the application of Shigeki, as well as changes in resource availability and community structure [28]. In contrast, *Basidiomycota*, *Mortierellomycota*, and *Rozellomycota* exhibited relatively stable, although variable, abundances across the analyzed soil samples. This pattern may indicate moderate sensitivity to environmental changes and an important role in maintaining soil ecosystem functioning. In particular, *Basidiomycota* are recognized as important decomposers of complex organic compounds. Under contaminated conditions, their ligninolytic and oxidoreductive enzymes facilitate the transformation of recalcitrant organic matter and chemical pollutants [29]. In the present study, the increased abundance of *Basidiomycota* in the PA_Am treatment may indicate a stimulatory effect of the Aminoprim biostimulant on enzymatic processes. Biostimulants have been reported to enhance the metabolic activity of saprotrophic fungi and promote the degradation of organic matter [30,31,32]. Furthermore, the increased abundance of *Mortierellomycota* and *Rozellomycota* in the PA_Am treatment may reflect a shift toward opportunistic organisms with a high capacity to adapt to changing habitat conditions and substrate availability. These groups are frequently associated with soils characterized by heterogeneous nutrient resources and disrupted competitive interactions, conditions that may favor their colonization of ecological niches emerging under environmental stress [33,34,35,36].

At the genus level, fungal community structure changed, with a decrease in the relative abundance of *Penicillium* and *Talaromyces* and an increase in *Humicola*, *Chaetomium*, and *Fusarium* in treatments amended with perfluorotetradecanoic acid and biostimulants. *Penicillium* and *Talaromyces* are typical colonizers of organic matter-rich environments and are well adapted to fluctuating conditions. However, their dominance may decline under intense chemical stress [30,32,33]. In contrast, *Humicola* and *Chaetomium* are saprotrophic fungi with extensive cellulolytic and hemicellulolytic enzyme systems that promote organic matter degradation, particularly in contaminated soils. The increased abundance of *Fusarium* may reflect its opportunistic nature and ability to rapidly colonize ecological niches emerging under disrupted microbial equilibrium and altered substrate availability [34]. Of particular ecological significance is the increased abundance of *Fusarium* in the PA_Am and PA_Sh treatments, as this genus includes both saprotrophic and potentially phytopathogenic species. Its proliferation may indicate weakened soil suppressiveness and destabilized antagonistic interactions among microorganisms. Such phenomena are frequently observed in soils exposed to long-term organic contamination, where microbial disturbances favor opportunistic taxa [6,37,38,39,40,41,42]. Furthermore, the increased relative abundance of *Fusarium* spp. in the PA_Am and PA_Sh treatments may indicate a substantial shift in the structure of the rhizosphere fungal community, particularly given that this genus includes numerous plant-pathogenic species. However, it should be emphasized that the genus *Fusarium* also comprises saprotrophic and endophytic species; therefore, increased abundance at the genus level cannot be directly associated with increased pathogen pressure. The increase in the relative abundance of these fungi may result from changes in the abundance of other microbial groups rather than from actual proliferation of *Fusarium* spp. [43]. Of particular relevance is the co-occurrence of an increased proportion of *Fusarium* spp. and reduced root biomass in the PA_Sh treatment. This pattern may indicate a potential interaction between PA-induced stress, root system condition, and changes in the rhizosphere microbiome. Stress and alterations in root development may modify the composition of root exudates and thereby select for specific groups of microorganisms. Conversely, the increased presence of *Fusarium* spp. cannot be ruled out as a contributing factor to the deterioration of root condition [44,45]. Therefore, the observed increase in *Fusarium* spp. should be regarded as a potential risk signal rather than direct evidence of the development of a pathogenic niche as a result of biostimulant application [43,44]. The increased contribution of the “*Others*” group in the PA_Sh and PA_Am samples indicates greater heterogeneity in fungal community structure under chemical stress. This observation is consistent with ecological theory, which predicts that environmental disturbances reduce the dominance of key taxa while promoting the persistence of rare microbial groups. Consequently, mycobiome complexity may increase through greater taxonomic diversity and a more even distribution of abundances, representing a typical response of soil ecosystems to anthropogenic pressure. However, such transformations may also reduce mycobiome functional stability by weakening ecological interaction networks and increasing the influence of stochastic processes on community assembly [34,46,47].

### 3.2. Alpha Diversity of Fungi in Soil Subjected to Perfluorotetradecanoic Acid and Biostimulant Treatments

The soil fungal alpha diversity revealed clear differences in community structure among the analyzed soil samples in the experiment, confirming previous reports on the significant influence of edaphic factors and land use on the soil microbiome [48,49]. The alpha diversity of soil fungi is primarily determined by soil properties (pH, organic carbon content, and soil structure), climatic conditions, and the availability of nutrients, which shape ecological niches and competition among taxa [50,51]. In the analyzed soil samples, the highest values of alpha diversity indices (Shannon, Simpson, Chao1, and the number of observed features) were recorded at the PA_Am treatment, indicating both high fungal richness and high community evenness. Greater heterogeneity of the soil environment and increased availability of different forms of organic matter can increase the number of available ecological niches, thereby limiting the dominance of individual taxa and promoting the coexistence of functionally diverse fungal groups [51,52]. Moreover, environments with higher microbial diversity are characterized by more stable soil ecosystem functioning, including more efficient nutrient cycling and greater resistance to disturbances [48].

In contrast, the PA and PA_Sh treatments exhibited lower alpha diversity and significantly higher dominance index values, indicating a simplified community structure and the predominance of a limited number of taxa. This phenomenon is commonly observed in environments subjected to stress or characterized by limited nutrient availability, where opportunistic microorganisms with a high adaptive capacity may be selectively favored. Under such conditions, decreased evenness and increased dominance may lead to a reduction in the overall complexity of the microbial community, potentially limiting its stability [53]. The observed increase in taxonomic richness accompanied by a decrease in fungal community evenness may result from the persistence or emergence of low-abundance taxa alongside an increase in the abundance of selected taxa better adapted to the altered environmental conditions. This pattern may reflect selective restructuring of the community, in which some taxa gain a competitive advantage while others remain present at low abundance. Rare taxa may actively influence community structure and modify the relative position of dominant taxa, and their composition may contribute to differences in microbiome responses to environmental changes [54,55]. In this context, the observed increase in the Chao1 index and the number of observed features, accompanied by decreases in the Shannon, Simpson, and Pielou indices, can be interpreted as a result of the selective expansion of certain taxa while rare taxa persist or emerge. This mechanism may explain the increase in community richness without a proportional increase in evenness and highlights the importance of both the characteristics of dominant taxa and their interactions with the rare biosphere in shaping microbial community structure [56]. The very high Good’s coverage values (0.998–1.000) confirm the high completeness of the sequencing effort, indicating that the results obtained provide a reliable representation of the fungal community structure. This is particularly important in metagenomic studies, as high sequencing coverage reduces the likelihood of underestimating rare taxa, which, despite their low abundance, may play important ecological roles in soil functioning [50,51].

### 3.3. Beta Diversity of Fungi in Soil Subjected to Perfluorotetradecanoic Acid and Biostimulant Treatments

Beta diversity analysis revealed significant differences among the studied treatments in both the taxonomic composition and phylogenetic organization of soil fungal communities [40,57,58]. The use of complementary distance metrics enabled a multidimensional assessment of differences among the soil samples. High Bray–Curtis dissimilarity values indicate substantial differences in the quantitative structure of fungal communities, primarily driven by differences in taxon dominance among treatments. In soil ecosystems, even small changes in abiotic factors, such as organic carbon content, moisture, or pH, cause pronounced shifts in community structure and in the dominance of specific microbial functional groups [59,60]. Higher Jaccard index values suggest greater similarity in fungal community taxonomic composition. These differences may reflect the influence of environmental conditions as well as limitations in microbial dispersal, both of which can contribute to the differentiation of fungal communities [61].

UniFrac analyses provided additional insights into fungal community phylogenetic structure. Unweighted UniFrac analysis indicated that differences among treatments were driven by turnover of deeply divergent evolutionary lineages. This suggests changes not only in taxon abundance but also in the composition of taxonomic groups. In contrast, weighted UniFrac values, which account for relative abundances, revealed substantial differences in the structure of dominant phylogenetic lineages. The wide range of UniFrac distances indicates that variability among treatments involved both the qualitative and quantitative components of the fungal communities. The PA_Sh treatment was particularly distinct, exhibiting the highest distance values. This indicates pronounced restructuring of the soil mycobiome, involving both dominant taxa and represented phylogenetic lineages. Such changes may reflect stronger selective pressures that narrow the ecological spectrum and favor specific fungal functional groups. Conversely, the high similarity between C_Sh and C_Am suggests a relatively stable fungal community structure under these conditions. This may reflect relatively homogeneous environmental conditions or similar mechanisms of microbial community assembly, resulting in comparable dominant taxa and stable abundance distributions [61,62,63,64,65,66,67].

Beta-diversity analysis revealed clear differences in fungal community structure among the analyzed soil samples, with a particularly distinct profile observed in PA_Sh. This treatment exhibited a pronounced shift in community structure toward *Chytridiomycota*, accompanied by a decrease in the relative abundance of *Ascomycota*, which dominated in the other treatments. Such a marked change in the proportions of the major fungal phyla indicates substantial reorganization of the fungal community structure in PA_Sh and is reflected in the beta-diversity analyses. The differences between PA_Sh and the other samples were particularly pronounced in the weighted UniFrac analysis. This metric accounts for both the phylogenetic relatedness of taxa and their relative abundances, making it particularly sensitive to changes in the representation of dominant phylogenetic lineages [68]. The observed pattern therefore suggests that the distinctiveness of PA_Sh is attributable not only to differences in taxonomic composition but also to shifts in the relative abundance of taxa belonging to different phylogenetic lineages. The high Jaccard distance values further indicate pronounced differences in taxonomic composition between PA_Sh and the other treatments. Consideration of both metrics indicates that the distinctiveness of PA_Sh reflects differences in both community composition and the relative abundance of individual taxa. Comparison of PA_Sh with C_Am further indicates that the presence of *Chytridiomycota* alone is insufficient to explain the observed pattern. Despite the increased relative abundance of *Chytridiomycota* in C_Am soil, the structure of this community remained more similar to that of the other treatments than to PA_Sh. This indicates that the presence of *Chytridiomycota* alone was not sufficient to account for the observed differences in fungal community structure. The pronounced distinctiveness of PA_Sh may therefore result from the combined effects of changes in community composition and structure, including an increased relative abundance of *Chytridiomycota*, a decreased relative abundance of *Ascomycota*, and shifts in the representation of other taxa. This is consistent with the current understanding of fungal community beta diversity as reflecting the interplay between differences in taxonomic composition and ecological processes shaping local communities, whose relative importance may depend on environmental conditions and the characteristics of individual microbial groups [69,70].

### 3.4. Predictive Trophic Characteristics of Fungi in Soil Subjected to Perfluorotetradecanoic Acid and Biostimulant Treatments

The dominance of saprotrophic fungi across all analyzed soil samples is consistent with their fundamental role in organic matter decomposition and nutrient cycling in terrestrial ecosystems [30,32,64,65]. The dominance of saprotrophic fungi may have been associated with the physicochemical properties of the soil used in the experiment, which may have favored the development of this trophic group. Factors such as pH, organic matter content, and nutrient availability can significantly influence fungal community structure and the relative abundance of individual trophic groups. Studies conducted across different soil systems have demonstrated associations between soil properties and the occurrence of specific fungal functional groups, including saprotrophs [16,71]. Therefore, the observed proportion of saprotrophic fungi may not be attributable solely to the presence of perfluorotetradecanoic acid (PA) but may also partly reflect the specific properties of the soil used in the experiment. At the same time, soil properties, particularly organic matter content and mineral fraction, may influence the sorption and bioavailability of PFASs and thereby modify their effects on microorganisms [72]. The fungal community response observed in the present study may therefore result from interactions between the initial soil properties and the presence of perfluorotetradecanoic acid, rather than solely from the effects of this compound. This is particularly relevant because the soil was collected from an *Amaranthus cruentus* cultivation site, and its properties may differ from those of other agricultural soils [16]. The substantial contribution of the Undefined Saprotroph group, particularly in the C, C_Sh, and PA, may indicate intensive decomposition and high availability of organic substrates favoring fungi with broad enzymatic capabilities [48]. The exceptionally high proportion of taxa unassigned to a trophic function, particularly in PA_Sh (80.34%), highlights current limitations in the functional characterization of soil fungi. As noted by Tedersoo et al. [64], a considerable fraction of fungal diversity remains functionally uncharacterized because of incomplete taxonomic resolution and the broad ecological versatility of many fungal taxa. The large proportion of this group may also indicate the presence of rare or poorly characterized evolutionary lineages associated with altered habitat conditions. The differences among treatments, particularly the increased abundance of multifunctional trophic groups in PA_Am (including Plant Pathogen–Soil Saprotroph–Wood Saprotroph and Undefined Saprotroph–Wood Saprotroph), highlight the role of opportunistic trophic strategies. Fungal trophic multifunctionality is generally interpreted as an expression of their ability to adapt to fluctuating environmental conditions and substrate availability. Such communities are often characteristic of habitats with dynamic organic matter composition and diverse resource pools, where ecological flexibility provides a competitive advantage [64,65,72,73].

Mycorrhizal fungi occurred at very low abundance across all analyzed soil samples, including ectomycorrhizal fungi (0.02–0.17%), ericoid mycorrhizal fungi (0.17–1.19%), and arbuscular mycorrhizal fungi (<0.01%). This community structure may indicate a limited role of plant–fungal symbioses, as commonly observed in environments with high nutrient availability or intensive organic matter mineralization [31,74]. The low abundance of arbuscular mycorrhizal fungi may also reflect limited host availability or soil conditions unfavorable for their development [31,75,76,77].

The dominance of saprotrophic fungi and the low abundance of mycorrhizal and pathogenic organisms suggest intensive nutrient cycling, with organic matter decomposition and mineralization playing central roles [78,79,80].

### 3.5. Response of Amaranthus cruentus to Perfluorotetradecanoic Acid and Biostimulant Treatments

The present study demonstrated that exposure of *Amaranthus cruentus* to perfluorotetradecanoic acid inhibited plant growth and development. Although the mechanisms underlying the phytotoxicity of long-chain perfluorocarboxylic acids remain incompletely understood, increasing evidence indicates that PFASs disrupt several plant physiological processes, including photosynthesis, hormonal regulation, nutrient metabolism, and cellular redox homeostasis. These effects can suppress plant growth and reduce biomass production [81,82,83]. PFAS phytotoxicity is commonly associated with oxidative stress. Excessive production of reactive oxygen species (ROS) can damage cellular membranes, impair chloroplast function, and reduce photosynthetic efficiency. Exposure may also alter antioxidant enzyme activity and intensify lipid peroxidation, ultimately impairing plant growth. The responses of *A. cruentus* observed in this study may also be related to the specific properties of long-chain perfluorotetradecanoic acid. Furthermore, these compounds can be absorbed by roots and subsequently accumulate in plant tissues. Such accumulation may interfere with water and nutrient uptake and disrupt vascular system function, ultimately inhibiting growth [84]. No significant changes in SPAD values were observed in the present experiment, which may indicate relatively stable chlorophyll content in the leaves despite exposure to PA. Similar results were reported by Valente et al. [85], who found no significant reduction in SPAD values in maize, sunflower, and hemp exposed to PFOA and PFOS, despite changes in gas exchange parameters and photosystem II functioning in hemp. This indicates that stable chlorophyll content does not necessarily imply the complete preservation of photosynthetic activity. In contrast, Lintern et al. [86] reported no significant changes in chlorophyll content or photosystem II efficiency in *Azolla filiculoides* exposed to seven PFASs. However, the SPAD response may depend on the plant species and PFAS dose. Alaoui et al. [87] found no changes under low-level PFOA exposure, whereas high concentrations reduced SPAD values in wheat and pea. Therefore, the absence of significant changes in SPAD values in the present study should be interpreted as an absence of a pronounced response in terms of chlorophyll content rather than as evidence of an absence of PFAS-induced stress. These compounds may affect plant metabolism and photosynthetic processes without causing pronounced changes in pigment content [88].

The application of biostimulants resulted in different plant responses. The beneficial effects of biostimulants on *A. cruentus* growth in treatments without perfluorotetradecanoic acid may have resulted from biologically active compounds in these preparations, including phytohormones, amino acids, polysaccharides, and other bioactive substances. These compounds can stimulate root system development, enhance nutrient uptake, and improve photosynthetic performance, thereby promoting increased biomass production. Biostimulants may also activate plant defense mechanisms by regulating antioxidant enzymes and strengthening ROS detoxification systems [80]. However, the results indicate that biostimulant effectiveness was limited under perfluorotetradecanoic acid-induced stress. This may be related to the high persistence and bioaccumulation potential of perfluorotetradecanoic acid, which can disrupt essential metabolic processes. Under severe chemical stress, biostimulant-induced activation of plant defense mechanisms may be insufficient to restore normal physiological function and growth [88,89]. The effects of the biostimulants on SPAD values varied depending on both the type of preparation and the presence of perfluorotetradecanoic acid. Shigeki did not cause significant changes in SPAD values in control soil (C), whereas in PA-amended soil, it resulted in a decrease in SPAD values. A different response was observed following the application of Aminoprim, which increased SPAD values under both experimental conditions. The increase in SPAD values following Aminoprim application may indicate a beneficial effect of this preparation on maintaining or increasing chlorophyll content in the leaves. This is consistent with recent studies indicating that amino acid-based biostimulants and protein hydrolysates may stimulate chlorophyll synthesis, improve mineral nutrient utilization, and support the functioning of the photosynthetic apparatus [90]. The differential plant response to Shigeki may indicate that its effects depend on the presence of a stress factor. Differences in mineral nutrient availability, pH, organic matter content, and soil contaminant levels may modify the effectiveness of the preparation and its effects on plant physiology. Similar variability in the effects of different biostimulants was reported by Gendaszewska et al. [91], who observed both increases and decreases in chlorophyll content depending on the preparation applied and its combination with other treatments.

The very low abundance of arbuscular mycorrhizal fungi (AMF < 0.01%) observed in the present study may also be relevant to plant tolerance to stress associated with soil contamination with perfluorotetradecanoic acid. AMF play an important role in plant responses to various abiotic stresses, including by increasing the availability of mineral nutrients, supporting root system development, and modulating antioxidant mechanisms. These functions may be particularly important under exposure to organic contaminants, which can disrupt metabolism, induce oxidative stress, and inhibit plant growth [92,93]. At the same time, such contaminants may adversely affect mycorrhizal fungi by limiting root colonization, hyphal development, and spore formation. This may result in a feedback mechanism in which contaminant pressure impairs the development of symbionts, while reduced mycorrhization limits the potential benefits that plants may derive from this symbiosis [94]. The protective effects of AMF may involve several interconnected mechanisms. Their extensive hyphal network increases the effective absorptive surface area of the root system, facilitating the uptake of phosphorus and other mineral nutrients and potentially helping to maintain plant metabolism under stress. In addition, mycorrhizal symbiosis may enhance the activity of antioxidant enzymes, such as superoxide dismutase, catalase, peroxidases, and ascorbate peroxidase, thereby limiting the accumulation of reactive oxygen species and associated oxidative damage. AMF may also modify rhizosphere properties and influence the activity of microorganisms involved in the transformation of organic contaminants [95].

The obtained results indicate that the plant response to the applied biostimulants was variable and depended on both the presence of the stressor and the type of preparation. Shigeki increased plant biomass in soil without PA, whereas in its presence, it resulted in a reduction in root biomass. Aminoprim, in contrast, inhibited root growth even in the absence of PA. These findings confirm that biostimulants do not act as universal growth promoters but may modify plant physiological and metabolic processes depending on the type of preparation, environmental conditions, and stress intensity [96]. The observed reduction in root biomass in the presence of PA may result from an interaction between the biostimulant and the contaminant. Biostimulants may modify rhizosphere properties, including pH, nutrient availability, root exudation, and microbial activity, thereby influencing plant responses to stress [97]. One possible explanation for the observed interaction between Shigeki and perfluorotetradecanoic acid may be a change in the bioavailability of this compound in the rhizosphere. Organic and microbial components of biostimulants may affect soil physicochemical properties and, consequently, the proportion of individual fractions of compounds available to plants. Substances derived from biostimulants may modify adsorption and desorption processes and shift the equilibrium between less and more bioavailable pools of compounds in the soil. The magnitude and direction of these changes depend largely on soil properties and the chemical characteristics of the material applied [98,99,100]. Biostimulants may also modify the composition and activity of microorganisms, which, under stress conditions, may have either beneficial or adverse effects on root development. Therefore, the involvement of potentially harmful microorganisms, including fungi of the genus *Fusarium*, cannot be excluded. The differential plant responses to Shigeki and Aminoprim also indicate that the effectiveness of biostimulants depends on their composition and mode of action. A positive effect of a preparation on total biomass does not necessarily imply stimulation of root growth, particularly under stress conditions. The effect of biostimulants on root system biomass depends on the type of preparation, plant species, and environmental conditions [100,101].

## 4. Materials and Methods

### 4.1. Experimental Design

The pot experiment was conducted in a vegetation hall at the University of Warmia and Mazury in Olsztyn using a completely randomized design with four replicates. The study included two experimental factors: the dose of perfluorotetradecanoic acid (0 and 0.16 µg kg^−1^ dry matter of soil) and the type of biostimulant (no biostimulant application, Shigeki at a rate of 2.5 dm^3^ ha^−1^, and Aminoprim at a rate of 3.5 dm^3^ ha^−1^). The model plant used in the experiment was Amaranthus cruentus. Plastic pots with a capacity of 3.0 dm^3^ were filled with 2.7 kg of soil. In the respective experimental treatments, perfluorotetradecanoic acid was applied once as an aqueous solution, ensuring its uniform distribution throughout the entire soil mass. Biostimulants were applied twice: on the day of experiment establishment and on day 25 of the experimental period. The control treatment consisted of soil without the addition of perfluorotetradecanoic acid and without biostimulant application. On the day of experiment initiation, the soil was supplemented with mineral nutrients, calculated per kilogram of soil dry matter, at the following rates: nitrogen—70 mg kg^−1^ as CO(NH_2_)_2_, phosphorus—30 mg kg^−1^ as KH_2_PO_4_, and potassium—100 mg kg^−1^ supplied as KH_2_PO_4_ and KCl. After preparation of the experimental setup, soil moisture was adjusted to 50% of capillary water capacity and maintained at this level throughout the entire experimental period. On day 50 of the experiment, soil samples were collected and used for analysis of the soil mycobiome using next-generation sequencing (NGS). After completion of the experiment, soil from four independent pots within each experimental treatment was pooled into a composite sample. The remaining plant root material was removed, and the soil was subsequently homogenized to obtain a uniform material representative of the respective experimental treatment. The prepared soil material was placed in sterile containers and stored at −32 °C until analysis. DNA was extracted from the pooled samples in three technical replicates for each experimental treatment. A separate sequencing library was prepared from each DNA isolate, and all libraries were subsequently subjected to sequencing. In addition, plant height, the biomass of the aboveground parts and roots of *A. cruentus*, and the SPAD leaf greenness index were determined. SPAD measurements were performed using a KONICA MINOLTA chlorophyll meter (Spectrum Technologies Inc., Chiyoda, Japan).

The experimental design is presented in Figure 13, while the abbreviations used for the experimental treatments are explained in Table 3.

### 4.2. Soil Properties and Characteristics

Before establishing the pot experiment, soil material was collected from arable land at a depth of 0–20 cm in Tomaszkowo (53°43′5.954″ N, 20°24′28.86″ E), located in the Warmian–Masurian Voivodeship in north-eastern Poland. The soil samples were then transported to the Didactic and Experimental Facility of the University of Warmia and Mazury in Olsztyn (53°46′36.062″ N, 20°28′35.425″ E), where further preparation procedures were conducted. The collected soil material was sieved through a 5 mm mesh to obtain a homogeneous fraction and to remove plant residues, roots, and skeletal fractions. This procedure was aimed at homogenizing the experimental material and ensuring its standardization before the initiation of the experiment. The study was conducted using soil classified as Eutric Cambisols [102], developed from sandy loam. The soil was selected for the study because Eutric Cambisols are among the most widely distributed soil types in Poland [103]. The basic physicochemical properties of the soil comprised pH, hydrolytic acidity, the sum of exchangeable base cations, cation exchange capacity, base saturation of the sorption complex, organic carbon content, and total nitrogen content. These parameters were determined according to the methodology described by Wyszkowska et al. [104,105]. Detailed soil characteristics are presented in Table 4.

### 4.3. Properties and Characteristics of Perfluorotetradecanoic Acid

In the present study, perfluorotetradecanoic acid (PA) was used in the form of the analytical standard PESTANAL^®^ (CAS No. 376-06-7, EC No. 206-803-4, purity ≥96.0%), purchased from Sigma-Aldrich Production GmbH (Buchs, Switzerland). The dose of perfluorotetradecanoic acid (0.16 µg kg^−1^ dry matter of soil) was established based on literature data [106,107,108,109], according to which PFAS concentrations in soils range from 0.001 ng g^−1^ to several µg g^−1^ dry matter of soil. Given the high chemical persistence of long-chain PFASs and their ability to undergo sorption and long-term retention in soil, even relatively low levels of exposure may affect biological processes and the physicochemical properties of the soil environment. Perfluorotetradecanoic acid belongs to the group of long-chain per- and polyfluoroalkyl substances (PFASs) and is characterized by the chemical structure CF_3_(CF_2_)_12_COOH, in which a fully fluorinated carbon chain is terminated by a carboxyl group. This molecular structure determines its exceptional thermal, chemical, and biological stability, as well as its high resistance to environmental degradation processes. The compound occurs in a solid state, with a molecular weight of 714.11 g mol^−1^. Perfluorotetradecanoic acid is characterized by a relatively low density ranging from 0.89 to 1.8 g cm^−3^, a melting point of 130–135 °C, and a boiling point of approximately 270 °C. Its thermal properties also include a flash point ranging from 120 to 192 °C, indicating its stability under a broad range of environmental conditions. From a toxicological perspective, perfluorotetradecanoic acid exhibits moderate acute toxicity, with an oral LD_50_ value of 500.1 mg kg^−1^, while inhalation toxicity (for vapors) has been reported at 11.1 mg dm^−3^. Due to the presence of a highly fluorinated aliphatic chain, perfluorotetradecanoic acid exhibits strong hydrophobicity and a tendency for substantial sorption to soil organic matter. These properties contribute to its long-term persistence in soil environments and limited mobility, which are characteristic features of long-chain PFASs [110].

### 4.4. Characteristics of the Applied Biostimulants

To modify plant growth conditions and mitigate the potential effects of environmental stress, two biostimulants were applied: Shigeki and Aminoprim. The Shigeki biostimulant (Futureco Bioscience S.A., Barcelona, Spain) is a formulation based on an extract of the marine algae *Ascophyllum nodosum* (10%) and enriched with phosphorus (10% P_2_O_5_), potassium (10% K_2_O), boron (0.13%), molybdenum (0.05%), and micronutrients in the form of EDTA chelates (Cu, Fe, Mn). This product exhibits biostimulatory activity by promoting root system development and enhancing plant tolerance to abiotic stresses. According to the manufacturer’s recommendations, it is applied at a rate of 2.0–3.0 dm^3^ ha^−1^. Aminoprim (Intermag, Olkusz, Poland) is an organic biostimulant containing a mixture of free amino acids and short-chain peptides of natural origin. Its composition includes, among others, glycine (11.6%), proline (6.5%), alanine (5.8%), glutamic acid (5.5%), hydroxyproline (4.5%), as well as other amino acids present in lower proportions. The formulation supports plant metabolic processes, enhances resistance to abiotic stress, and accelerates recovery after the cessation of stress-inducing factors. The manufacturer-recommended application rate is 2.0–5.0 dm^3^ ha^−1^ of working solution.

### 4.5. Characteristics of Amaranthus cruentus

The model plant used in the present study was *Amaranthus cruentus*. It is an annual herbaceous species characterized by a C_4_ photosynthetic pathway and reproducing exclusively through sexual propagation via seeds. *A. cruentus* exhibits rapid growth and a short life cycle (approximately 4–6 weeks), enabling experimental studies to be conducted within a relatively short period. An important criterion for selecting this species was its high tolerance to abiotic stress factors, including drought, salinity, and the presence of chemical contaminants in soil. This adaptability allows *A. cruentus* to develop under conditions that may be unfavorable for many other plant species, making it a suitable model for investigating the effects of environmental stressors on plant growth and physiological responses. Additionally, this species develops an extensive root system, which promotes intensive interactions with soil microorganisms and enables the assessment of changes occurring in the structure and functioning of microbial communities within the rhizosphere [111,112].

*Amaranthus cruentus* is one of the three major amaranth species cultivated for seed production, along with *A. caudatus* and *A. hypochondriacus*. It has diverse applications as a pseudocereal, leafy vegetable, and forage crop. Increasing interest in this species is driven by its high nutritional value, short growing season, and ability to be cultivated under water-limited conditions. Seed production is its primary use, and the seeds can be consumed whole, popped, or processed into flour. They are characterized by a high protein content, a favorable amino acid profile, and the presence of unsaturated fatty acids, tocopherols, phytosterols, and squalene, which enhance their suitability for functional food production. Young leaves are used as a vegetable, while the remaining biomass can be utilized as animal feed. The diverse applications of *A. cruentus* increase its economic importance and its potential to support agricultural diversification and food security [113,114,115].

### 4.6. DNA Sequencing Analysis and Bioinformatic Processing of Data

The material used for microbiological analysis consisted of soil samples collected at the end of the plants’ growing season. Following soil collection, DNA was extracted from 0.5 g of fresh soil. For each experimental variant, soil was collected from four independent pots and pooled to form a single composite sample, which was treated as one biological sample. Three independent DNA extractions were performed from each pooled sample and treated as technical replicates. Each DNA extract was used to prepare a separate metagenomic library for sequencing. Thus, three metagenomic libraries were prepared for each experimental variant. Accordingly, the pooled soil sample represented the basic biological sample, while the individual pots contributed to the pooled sample and the DNA extraction replicates were not considered independent biological replicates. DNA isolation and bioinformatic analyses were performed at Novogene (UK) Company Limited (Cambridge, UK). Taxonomic identification of fungi was conducted based on the internal transcribed spacer (ITS) region, which was amplified using barcoded primers (Table 5). PCR amplification was carried out in a total reaction volume of 15 μL using Phusion High-Fidelity PCR Master Mix (Thermo Fisher Scientific, Waltham, MA, USA), primers at a concentration of 0.2 μM, and 10 ng of DNA template. The PCR products were purified, normalized, and pooled to generate sequencing libraries. The quality of the libraries was assessed using Qubit fluorometric (Thermo Fisher Scientific, Waltham, MA, USA) quantification, qPCR, and bioanalyzer-based analysis. Sequencing was performed using the NovaSeq 6000 platform (Illumina, San Diego, CA, USA). The obtained reads were assigned to individual samples based on index sequences and subsequently subjected to bioinformatic processing. Paired-end reads were merged using FLASH (v1.2.11), while sequence quality control was performed using fastp (v0.23.1). Chimeric sequences were removed using VSEARCH (v2.16.0) against the UNITE reference database. The final amplicon sequence variants (ASVs) were generated using QIIME2 (v2023.5) with the DADA2 algorithm. The results of the quality assessment of the obtained sequencing reads are presented in Table S2. The fungal sequence data generated in this study have been deposited in the National Center for Biotechnology Information (NCBI) database and are available under accession numbers PZ691301–PZ692160.

### 4.7. Data Processing and Statistical Analysis

The analysis of data concerning the structure and diversity of fungal communities was performed using bioinformatic, statistical, and graphical tools. All statistical analyses of the metagenomic data were conducted in the R environment (version 4.0.3), using, among others, the vegan (v. 2.7.3) and ggplot2 packages (v 4.0.2). The results were visualized using ChiPlot (https://chiplot.online/, accessed on 10 June 2026), SRplot (http://www.bioinformatics.com.cn/, accessed on 15 June 2026), and TBtools-II v 2.310 [116]. Principal Component Analysis (PCA) was performed using Statistica 13.3 [117].

Alpha diversity was assessed based on Observed Features, Good’s Coverage, Dominance, the Chao1 richness estimator, the Shannon and Simpson diversity indices, and Pielou’s evenness index. These indices enabled the assessment of taxonomic richness, diversity, and evenness across individual samples. Differences in alpha diversity indices among the analyzed groups were assessed using one-way analysis of variance (ANOVA) when the assumptions of normality of residuals and homogeneity of variance were met. When these assumptions were not satisfied, the non-parametric Kruskal–Wallis test was applied. Following a significant ANOVA result, multiple comparisons were performed using Tukey’s HSD test, whereas significant Kruskal–Wallis results were followed by pairwise comparisons using Dunn’s test.

Beta diversity, reflecting differences in fungal community structure among samples, was assessed based on Bray–Curtis and Jaccard distance matrices, as well as the phylogenetic UniFrac metrics, i.e., Unweighted and Weighted UniFrac. These distance matrices were used to perform Principal Coordinates Analysis (PCoA), enabling graphical representation of differences in community composition among samples. The statistical significance of differences in community structure among the analyzed groups was assessed using permutational multivariate analysis of variance (PERMANOVA) based on Bray–Curtis, Jaccard, Unweighted UniFrac, and Weighted UniFrac distance matrices. Statistical significance was assessed using 9999 permutations.

To identify taxa differing in relative abundance among the analyzed groups, differential abundance analyses were performed. For multiple hypothesis testing, *p*-values were adjusted using the Benjamini–Hochberg method to control the false discovery rate (FDR). An FDR < 0.05 was considered statistically significant.

The potential ecological functions of the fungal communities were characterized using the FUNGuild database (https://www.funguild.org/, accessed on 20 May 2026). This tool enables the predictive assignment of identified fungal taxa to specific trophic guilds based on their taxonomic information obtained from ITS-region sequencing data. The resulting assignments were used to characterize the predicted trophic structure of the analyzed fungal communities.

To assess potential relationships among fungal taxa, a co-occurrence analysis was performed at the genus level. Based on the identified relationships, a co-occurrence network was constructed, and its analysis and visualization were performed using Gephi version 0.10.1. The network analysis enabled the visualization of potential associations among individual fungal genera and the identification of their relationships within the analyzed communities.

In addition, correlation analysis was performed to assess the relationships between the relative abundances of dominant fungal taxa and the analyzed plant parameters. Pearson’s linear correlation coefficient (*r*) was used to assess the direction and strength of these relationships. This analysis enabled the assessment of potential associations between fungal community structure and plant growth and physiological parameters. The statistical significance of the correlation coefficients was assessed at α = 0.05.

## 5. Conclusions

The present study demonstrated that perfluorotetradecanoic acid (PA) significantly affected the taxonomic, phylogenetic, and functional structure of soil fungal communities. The compound induced a reorganization of the taxonomic structure of the mycobiome, manifested by changes in the abundance of dominant fungal phyla and genera. The most pronounced alterations involved fungi belonging to the phyla *Ascomycota* and *Chytridiomycota*, as well as the genera *Penicillium*, *Talaromyces*, *Humicola*, *Chaetomium*, and *Fusarium*. At the same time, perfluorotetradecanoic acid modified the alpha diversity of fungal communities, as reflected by decreased values of the Shannon, Simpson, Pielou, Observed features, and Chao1 indices, accompanied by an increase in the dominance index. These changes indicate a reduction in fungal diversity, taxonomic richness, and community evenness, together with an increased dominance of a limited number of taxa. The investigated compound also influenced beta diversity, causing alterations in taxonomic composition, quantitative community structure, and phylogenetic relationships within fungal assemblages, as confirmed by analyses based on Bray–Curtis dissimilarity, the Jaccard index, and UniFrac metrics. However, due to the replication structure of the experiment and the limited number of independent biological units, these results should be interpreted with caution. The predictive analysis suggested variation in the functional composition of the fungal communities; however, these findings should be interpreted with caution due to the relatively high proportion of sequences for which no functional assignment could be made. Biostimulant application modified the mycobiome response to perfluorotetradecanoic acid contamination. Among the tested formulations, Aminoprim was more effective than Shigeki at mitigating adverse changes in fungal community structure and diversity, promoting a more even community structure. The short-term pot experiment demonstrated that PA affected the growth of *Amaranthus cruentus* and the structure of soil fungal communities, whereas the application of biostimulants modified the plant and mycobiome responses to the presence of this compound in the soil. The interpretation of the obtained results should take into account the strictly controlled experimental conditions and the use of a single PA concentration, as well as the fact that PA residues in the soil and direct functional indicators of microbial processes were not analyzed in the present study. Therefore, long-term studies under field conditions, involving soils with different properties and a range of PA concentrations, as well as additional chemical and functional analyses, are necessary to expand our understanding in this area.

## Figures and Tables

**Figure 1:**
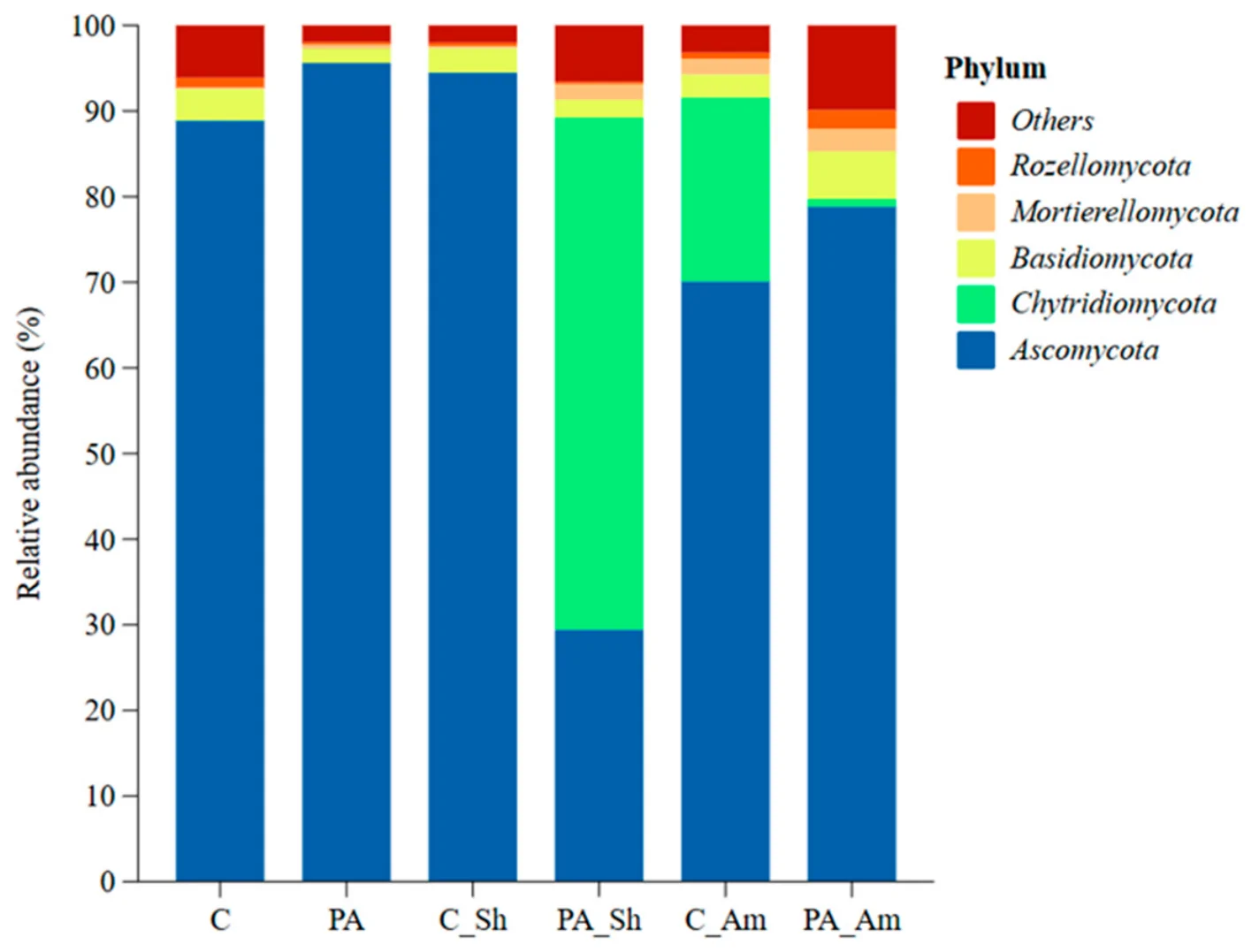
Dominant fungal phylum (%) in soil subjected to perfluorotetradecanoic acid and biostimulant treatments. C—soil without the addition of perfluorotetradecanoic acid and biostimulants; PA—soil supplemented with perfluorotetradecanoic acid; C_Sh—soil supplemented with the Shigeki biostimulant; PA_Sh—soil supplemented with perfluorotetradecanoic acid and the Shigeki biostimulant; C_Am—soil supplemented with the Aminoprim biostimulant; PA_Am—soil supplemented with perfluorotetradecanoic acid and the Aminoprim biostimulant.

**Figure 2:**
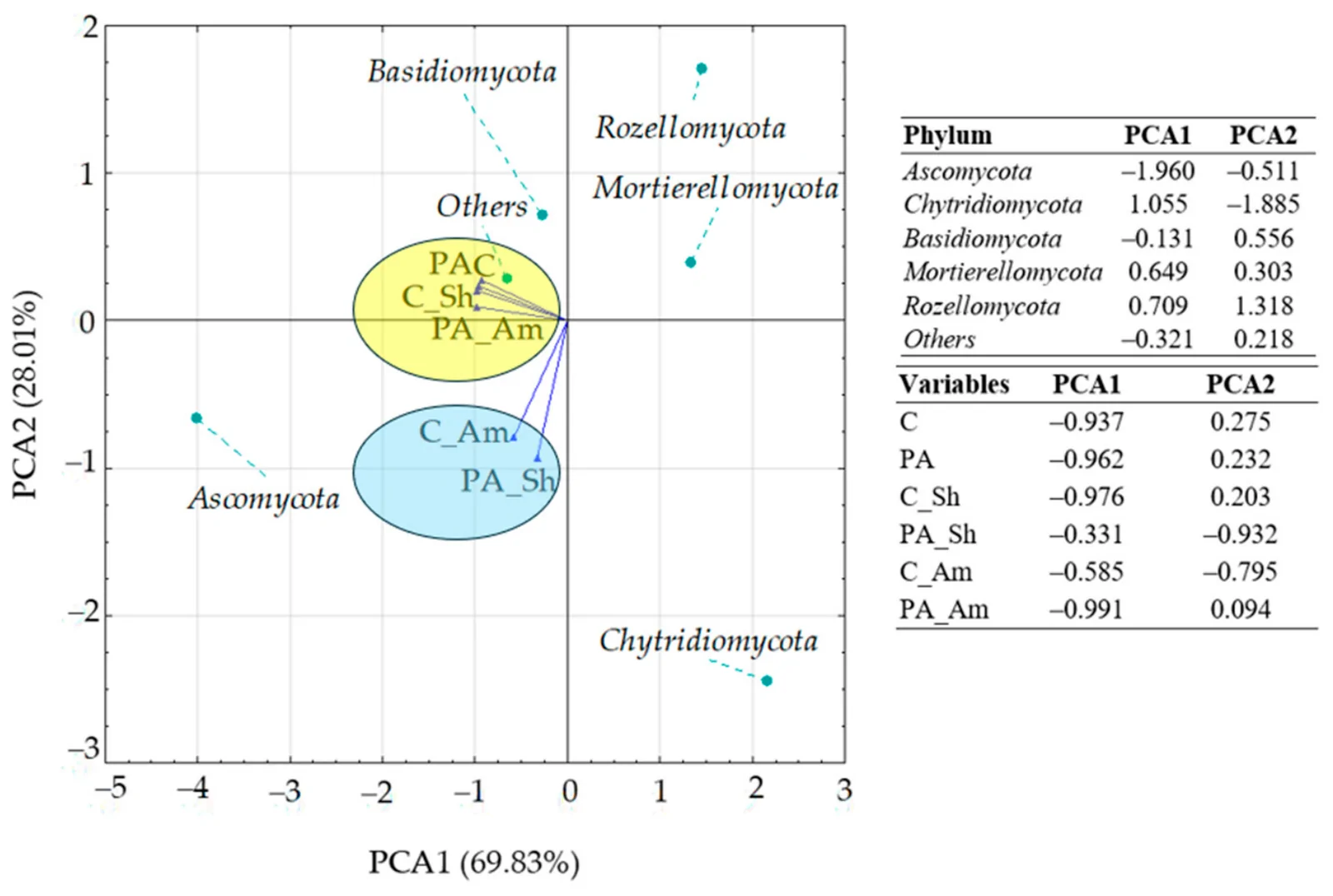
Principal component analysis (PCA) of changes in fungal community structure at the phylum level in soil subjected to perfluorotetradecanoic acid and biostimulant treatments. Abbreviations are explained in Figure 1.

**Figure 3:**
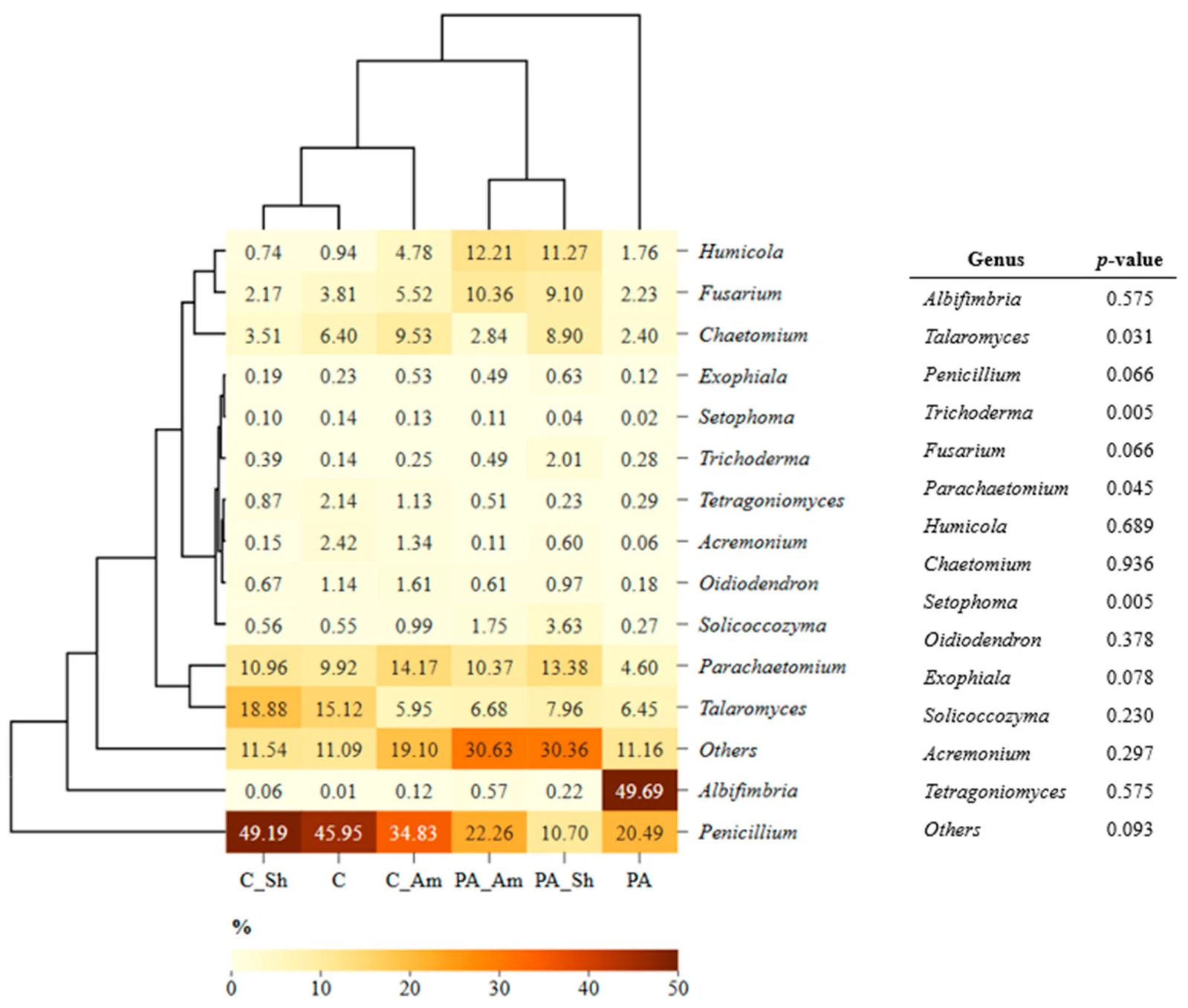
Dominant fungal genera (%) in soil subjected to perfluorotetradecanoic acid and biostimulant treatments. Abbreviations are explained in Figure 1.

**Figure 4:**
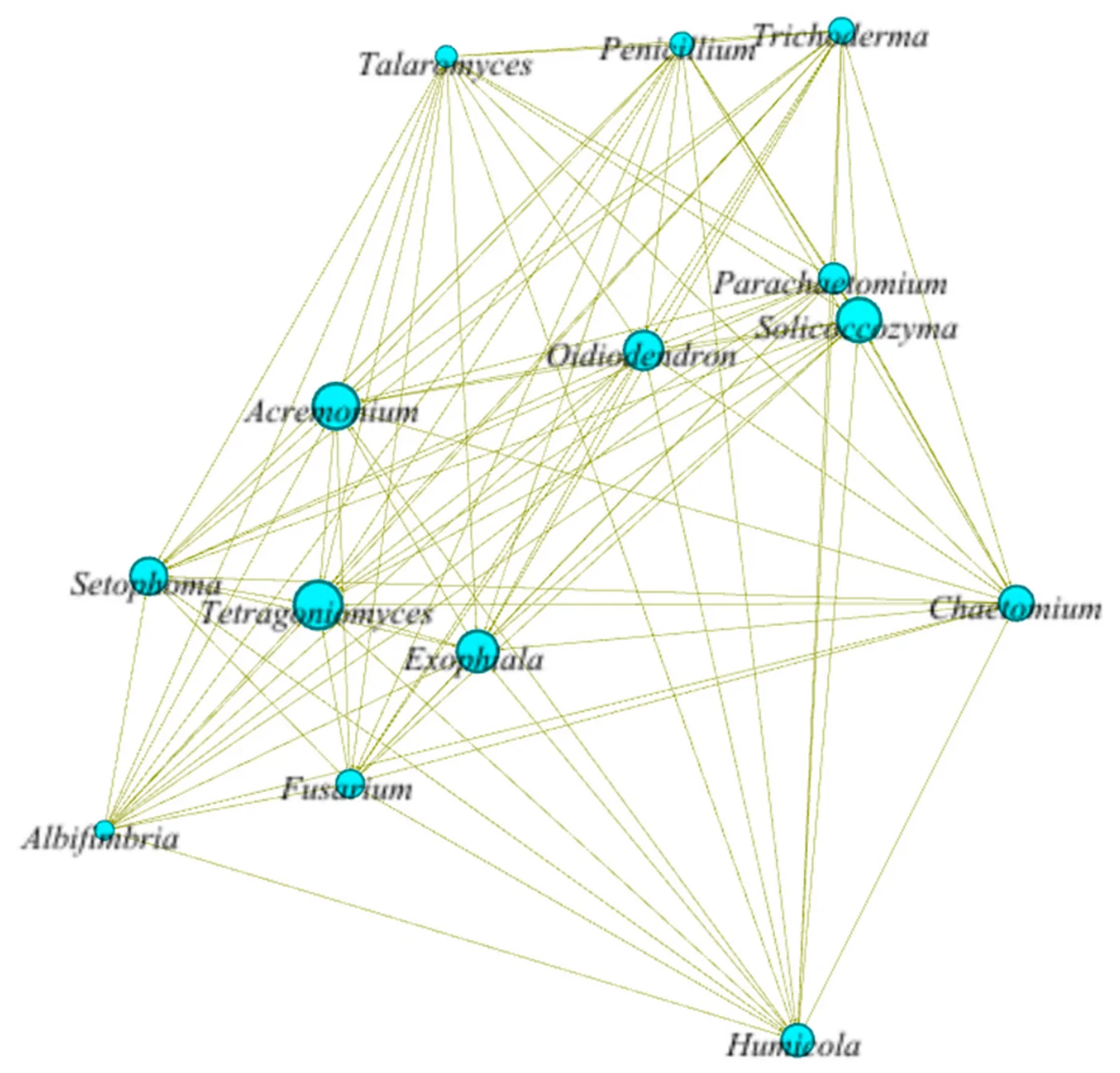
Co-occurrence network of the dominant fungal genera. Nodes represent individual fungal genera, with node size proportional to the number of connections with other taxa. Lines between nodes represent significant Pearson correlations between fungal genera (r > 0.6; *p* < 0.05).

**Figure 5:**
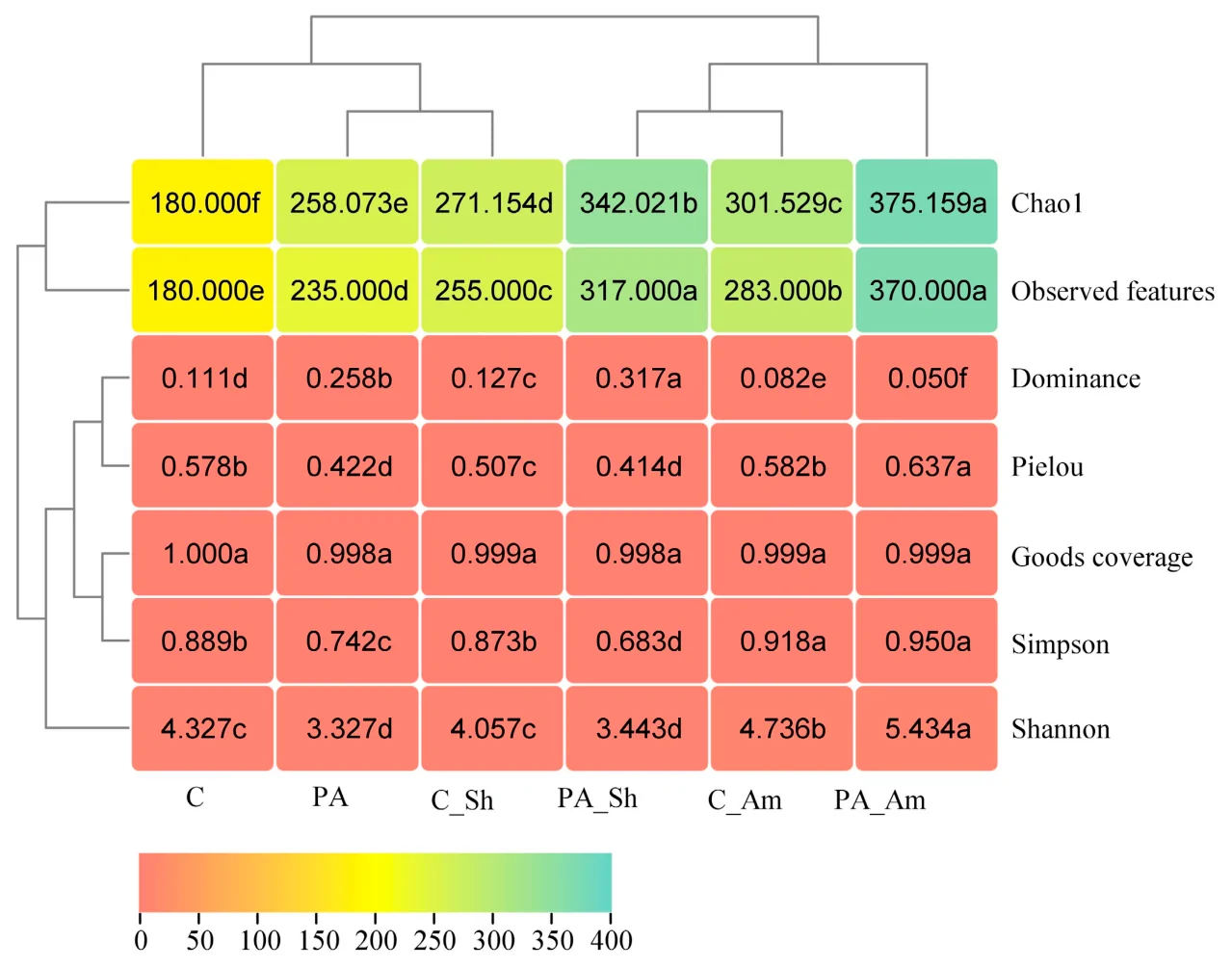
Alpha diversity of fungi in soil subjected to perfluorotetradecanoic acid and biostimulant treatments. Abbreviations are explained in Figure 1. Homogeneous groups indicated by different letters (a–f) were determined separately for each parameter.

**Figure 6:**
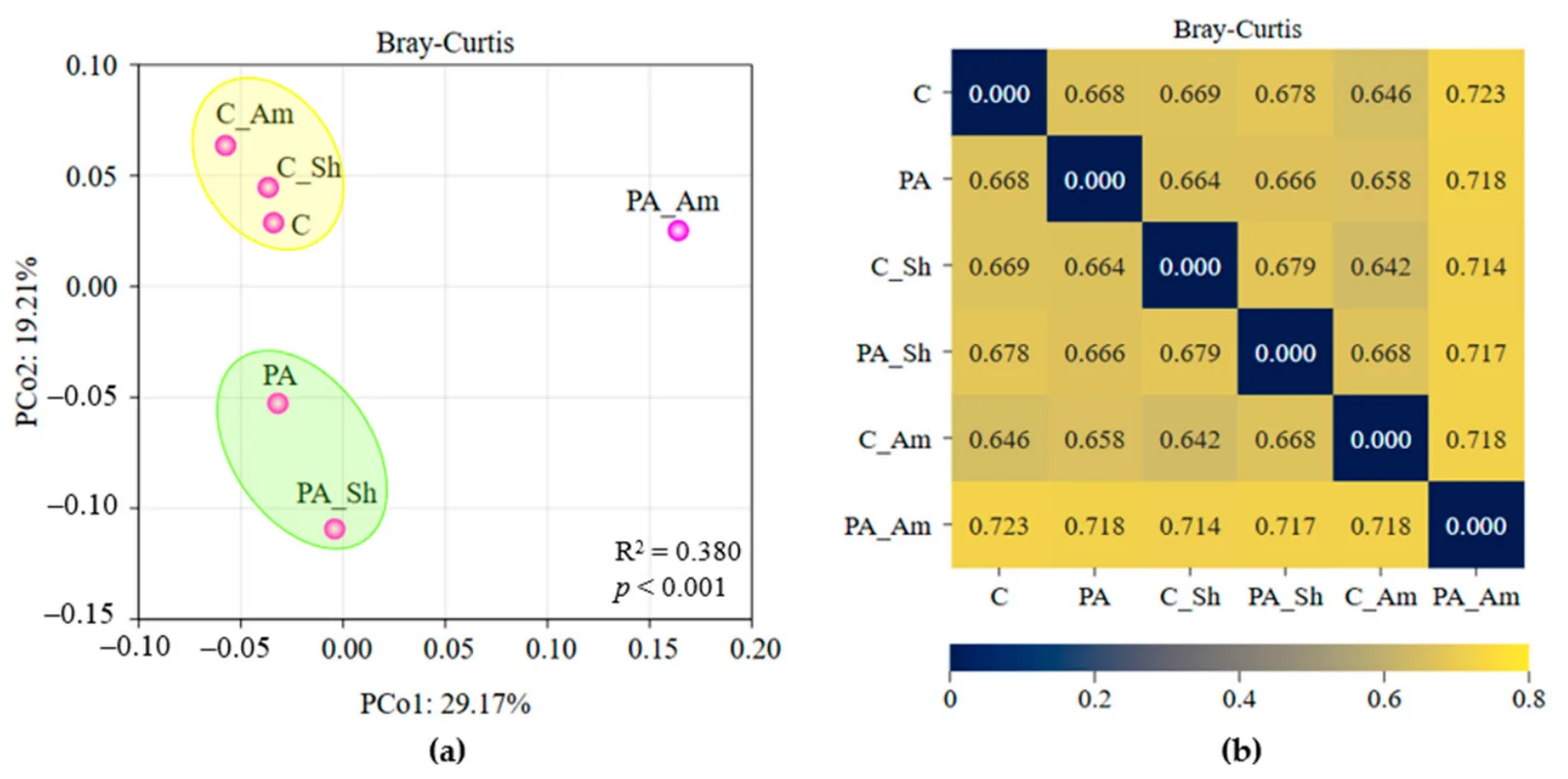
Beta diversity of fungal communities based on Bray–Curtis dissimilarity, presented as (**a**) Principal Coordinates Analysis (PCoA) and (**b**) a heatmap. Abbreviations are explained in Figure 1.

**Figure 7:**
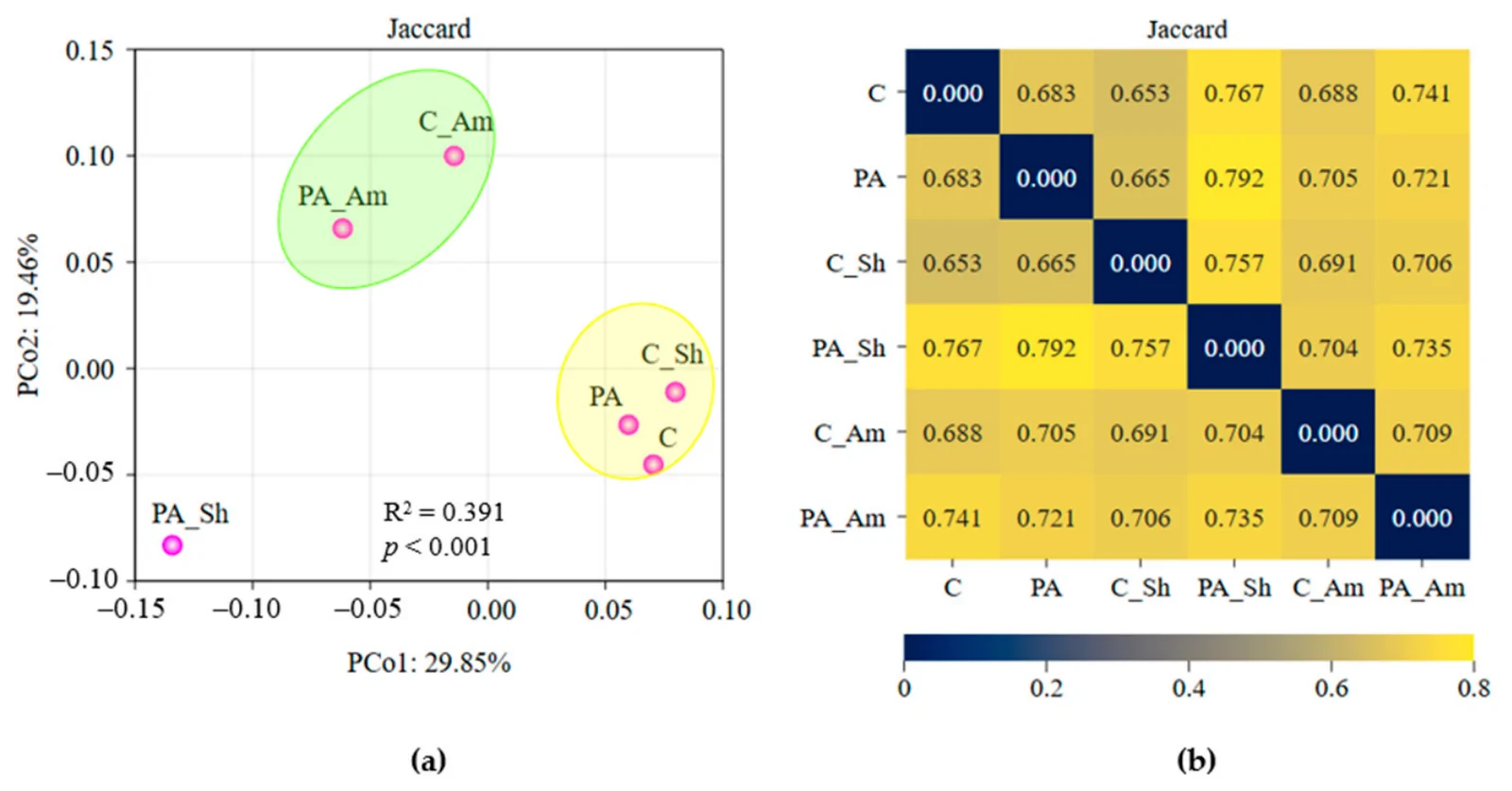
Beta diversity of fungal communities based on Jaccard dissimilarity, presented as (**a**) Principal Coordinates Analysis (PCoA) and (**b**) a heatmap. Abbreviations are explained in Figure 1.

**Figure 8:**
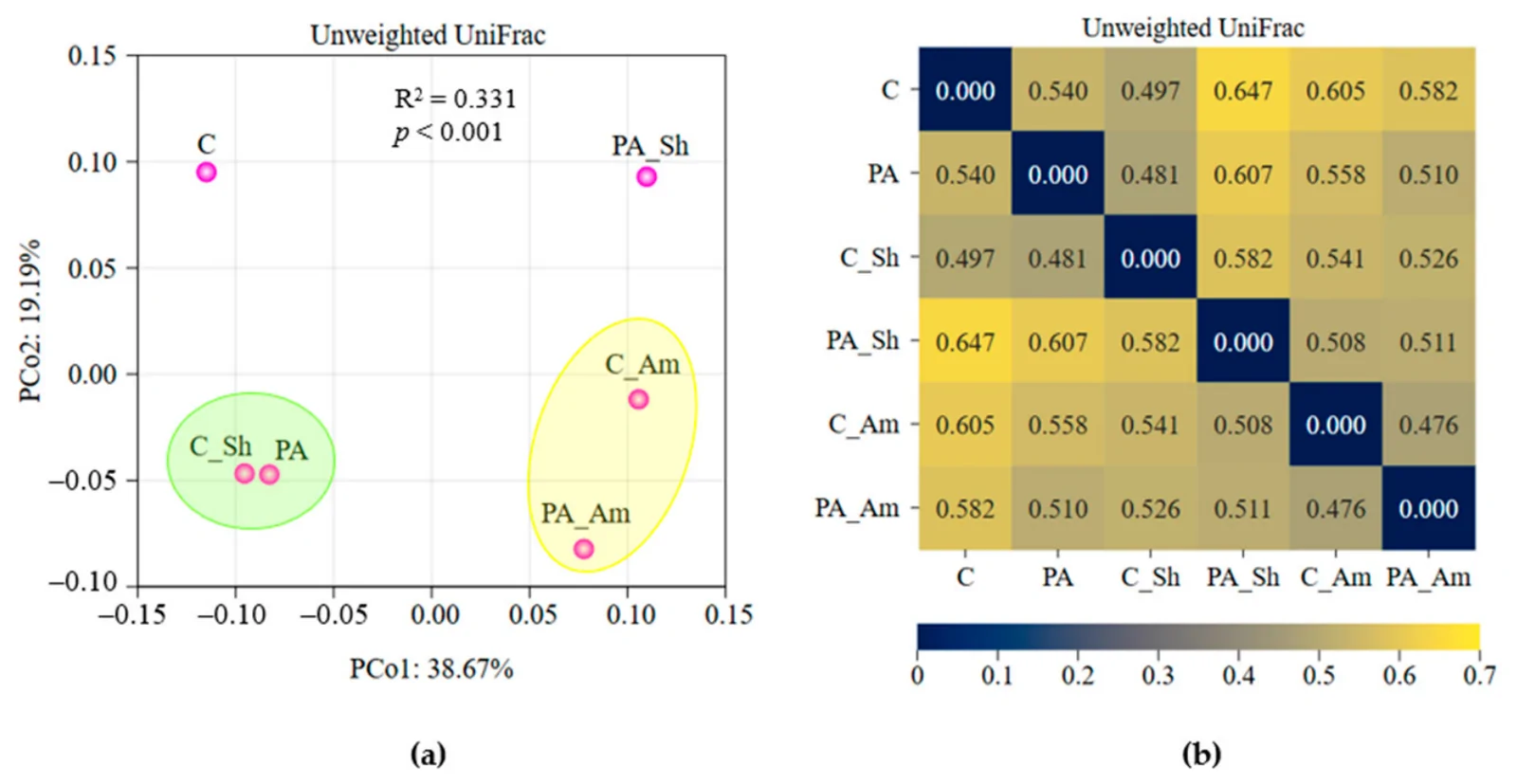
Beta diversity of fungal communities based on Unweighted UniFrac dissimilarity, presented as (**a**) Principal Coordinates Analysis (PCoA) and (**b**) a heatmap. Abbreviations are explained in Figure 1.

**Figure 9:**
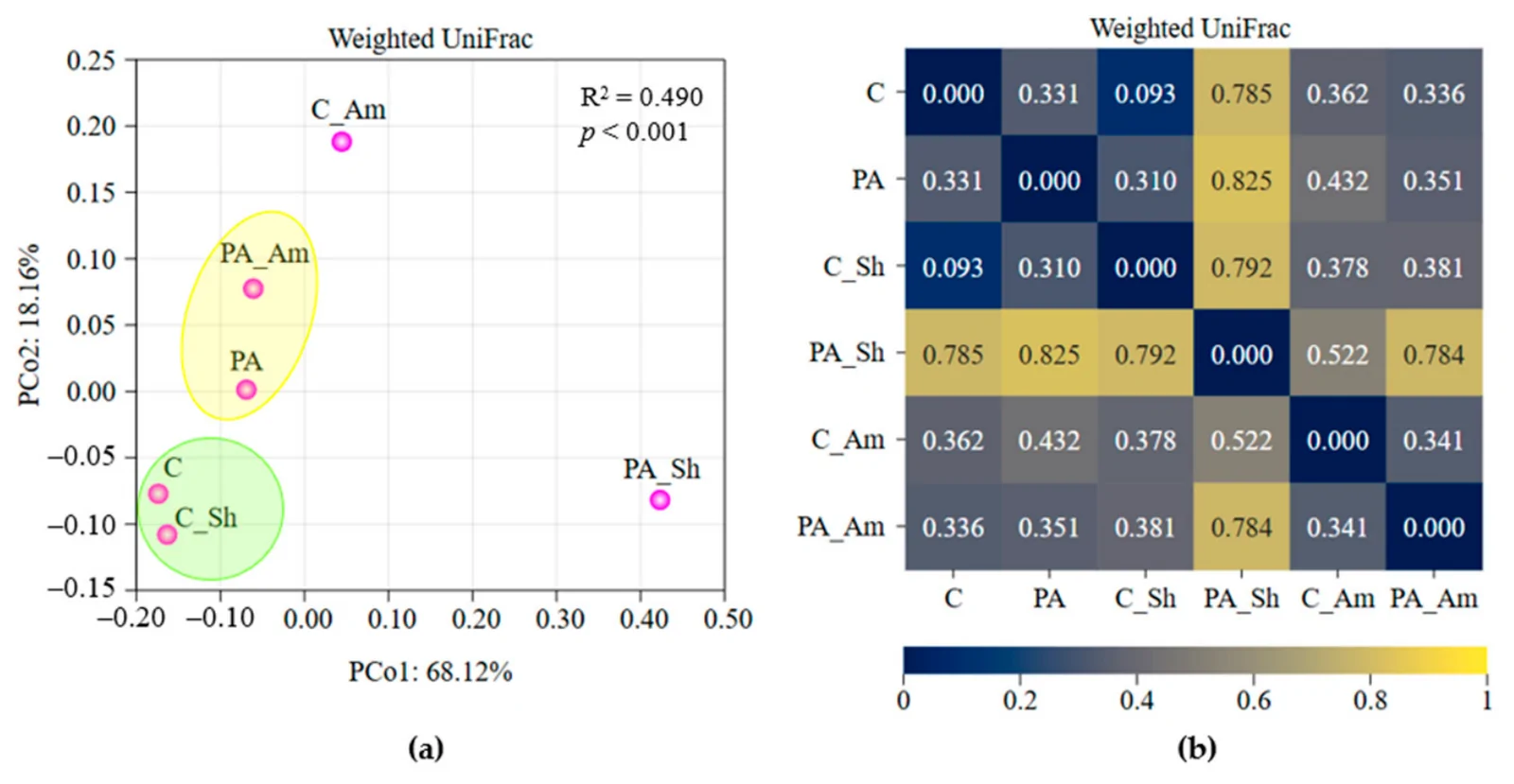
Beta diversity of fungal communities based on Weighted UniFrac dissimilarity, presented as (**a**) Principal Coordinates Analysis (PCoA) and (**b**) a heatmap. Abbreviations are explained in Figure 1.

**Figure 10:**
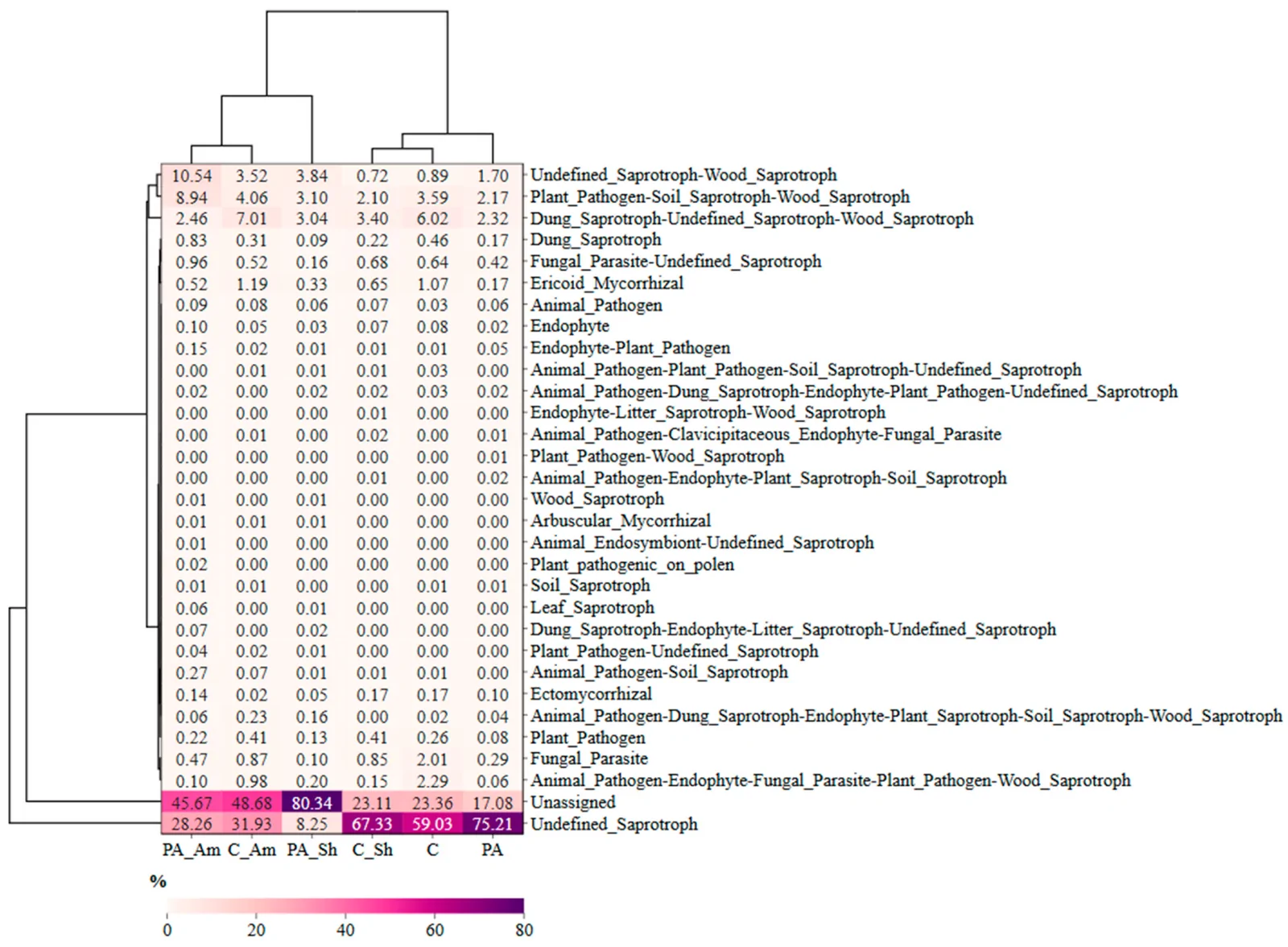
Potential trophic functions of fungi in soil subjected to perfluorotetradecanoic acid and biostimulant treatments. Abbreviations are explained in Figure 1.

**Figure 11:**
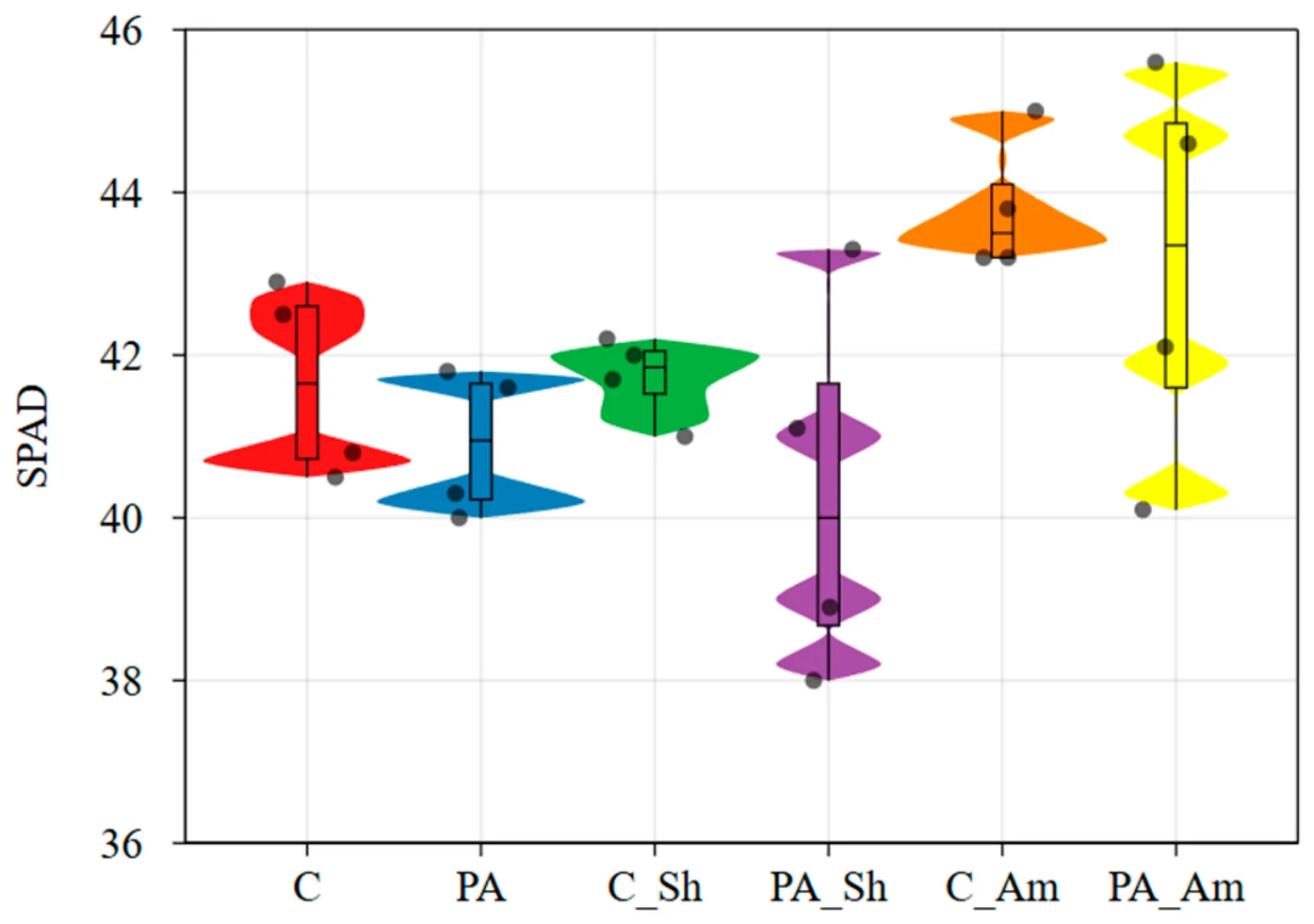
Leaf greenness index (SPAD) of *Amaranthus cruentus* grown in soil treated with perfluorotetradecanoic acid and biostimulants. Abbreviations are explained in Table 1.

**Figure 12:**
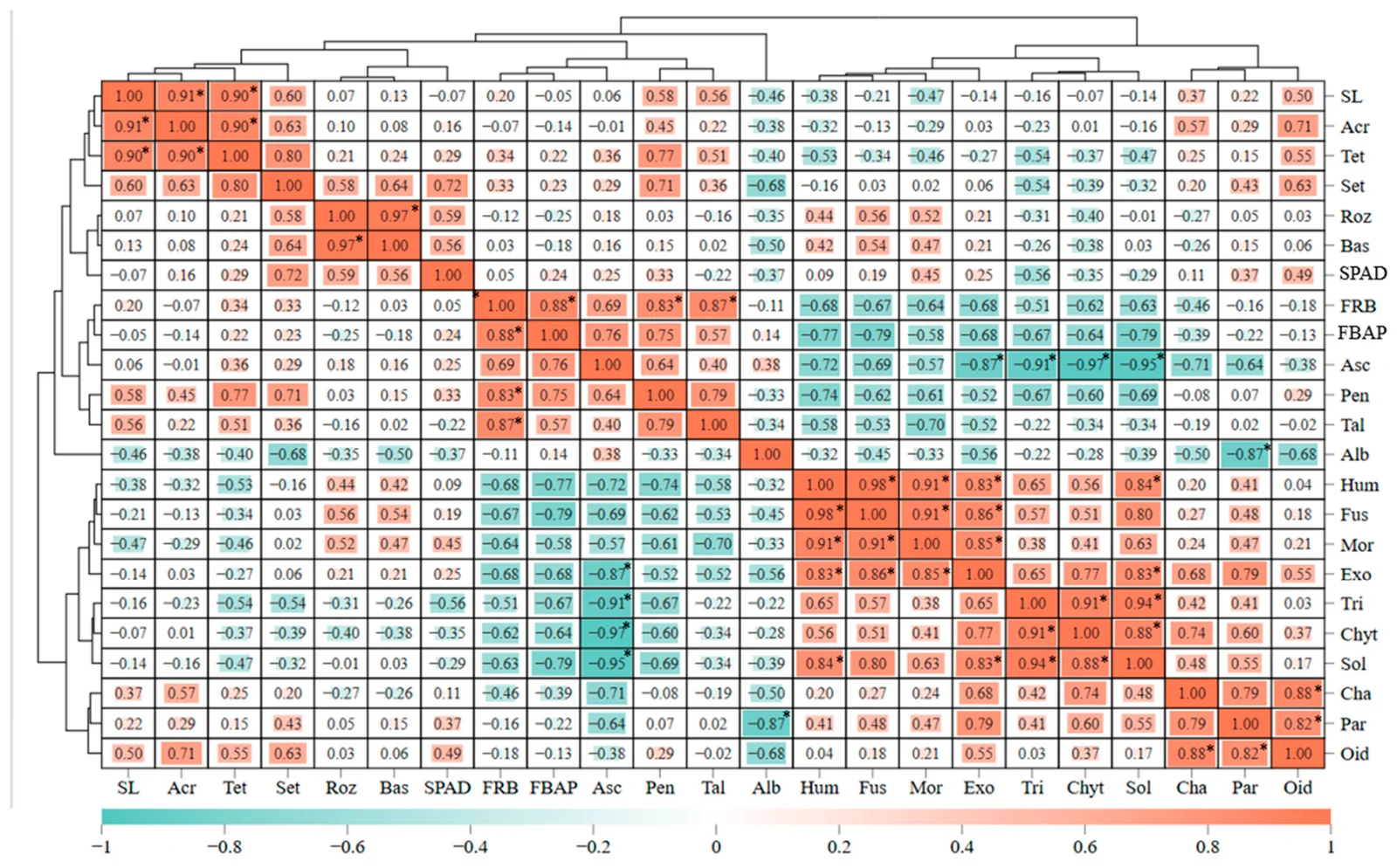
Pearson correlation coefficients between the structure of dominant fungal communities and selected growth parameters of *Amaranthus cruentus*. Asc—*Ascomycota*, Chy—*Chytridiomycota*, Bas—*Basidiomycota*, Mor—*Mortierellomycota*, Roz—*Rozellomycota*, Alb—*Albifimbria*, Tal—*Talaromyces*, Pen—*Penicillium*, Tri—*Trichoderma*, Fus—*Fusarium*, Par—*Parachaetomium*, Hum—*Humicola*, Chea—*Chaetomium*, Set—*Setophoma*, Oid—*Oidiodendron*, Exo—*Exophiala*, Sol—*Solicoccozyma*, Acr—*Acremonium*, Tet—*Tetragoniomyce*, SL—stem length; FBAP—fresh biomass of aboveground parts, FBRP—fresh biomass of root parts, SPAD—leaf greenness index. * statistically significant difference (*p* < 0.05).

**Figure 13:**
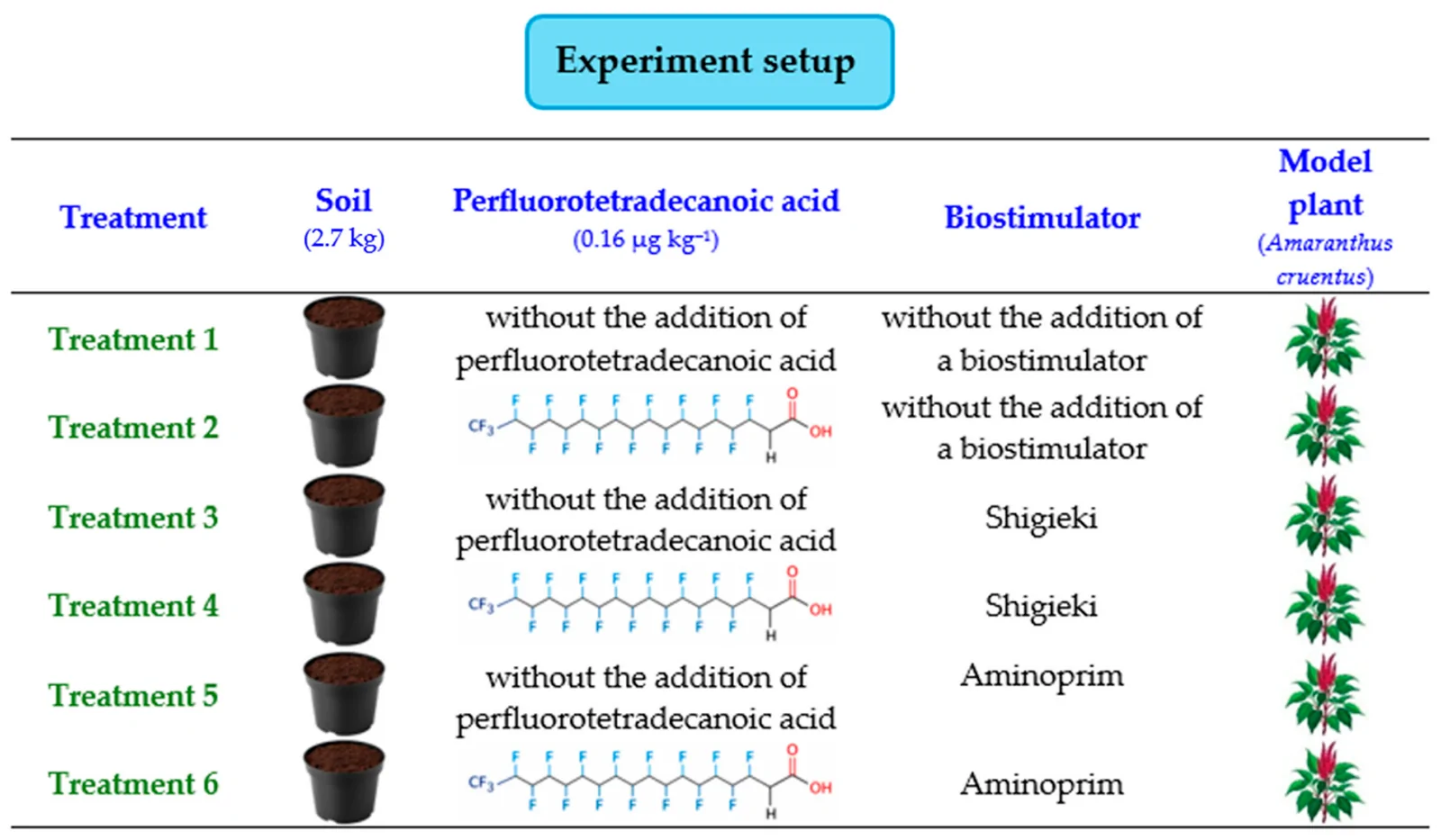
Schematic of the pot experiment design.

**Table 1:** Morphological parameters of the aboveground parts of *Amaranthus cruentus* at the BBCH 39 growth stage.

Parameter	Treatments					
	C	PA	C_Sh	PA_Sh	C_Am	PA_Am
	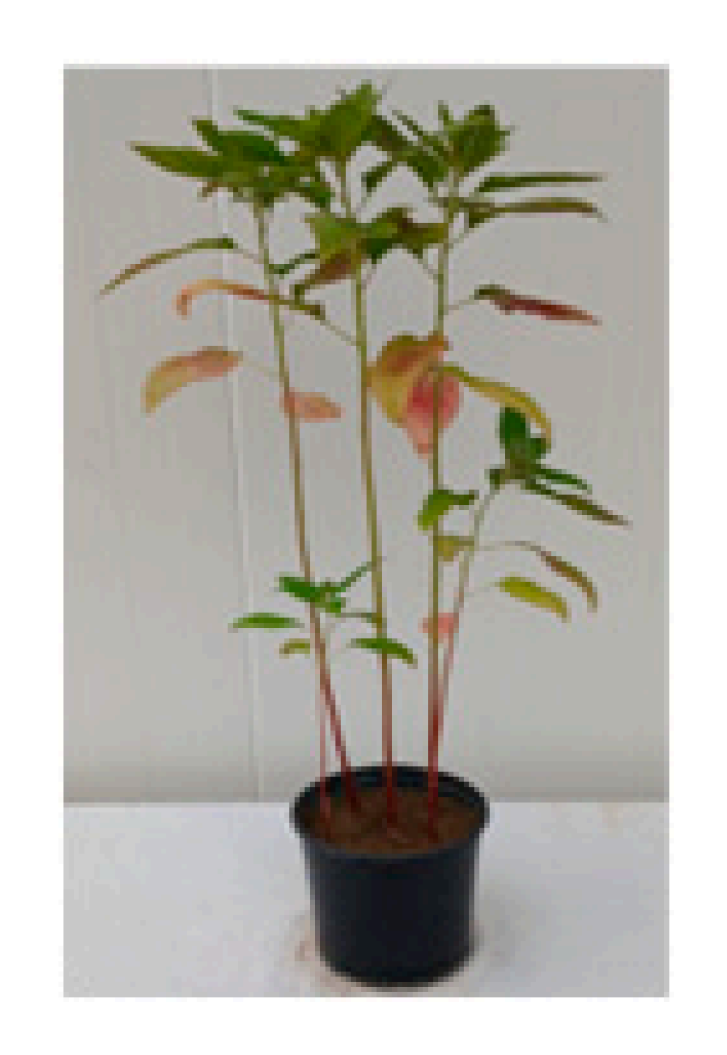	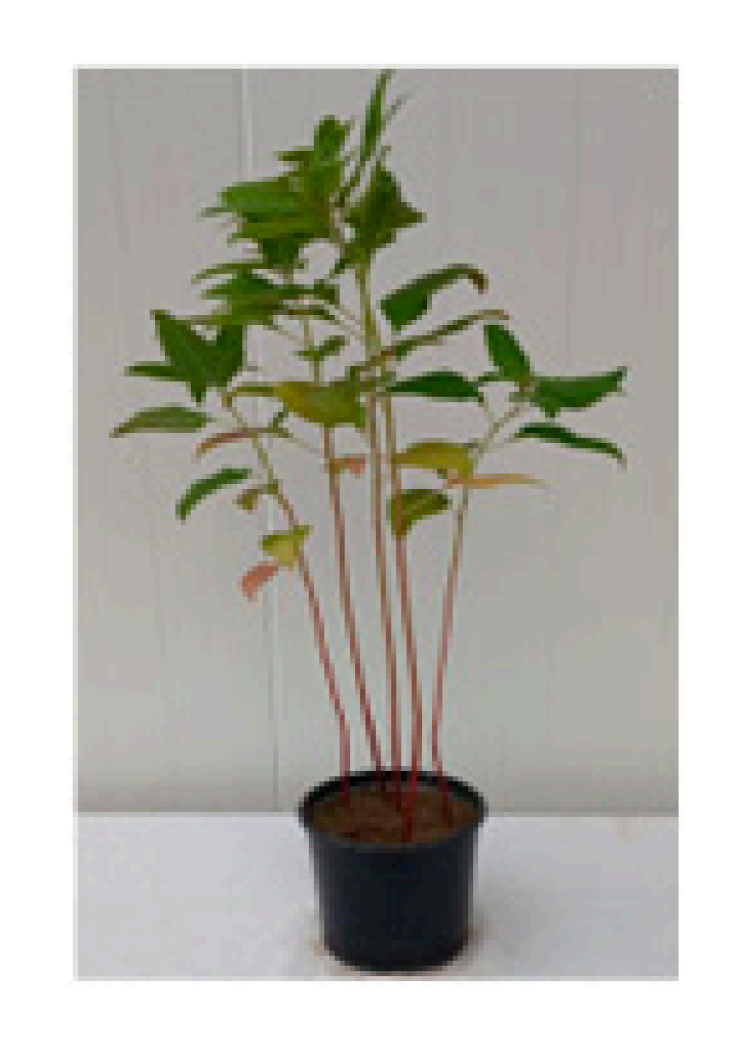	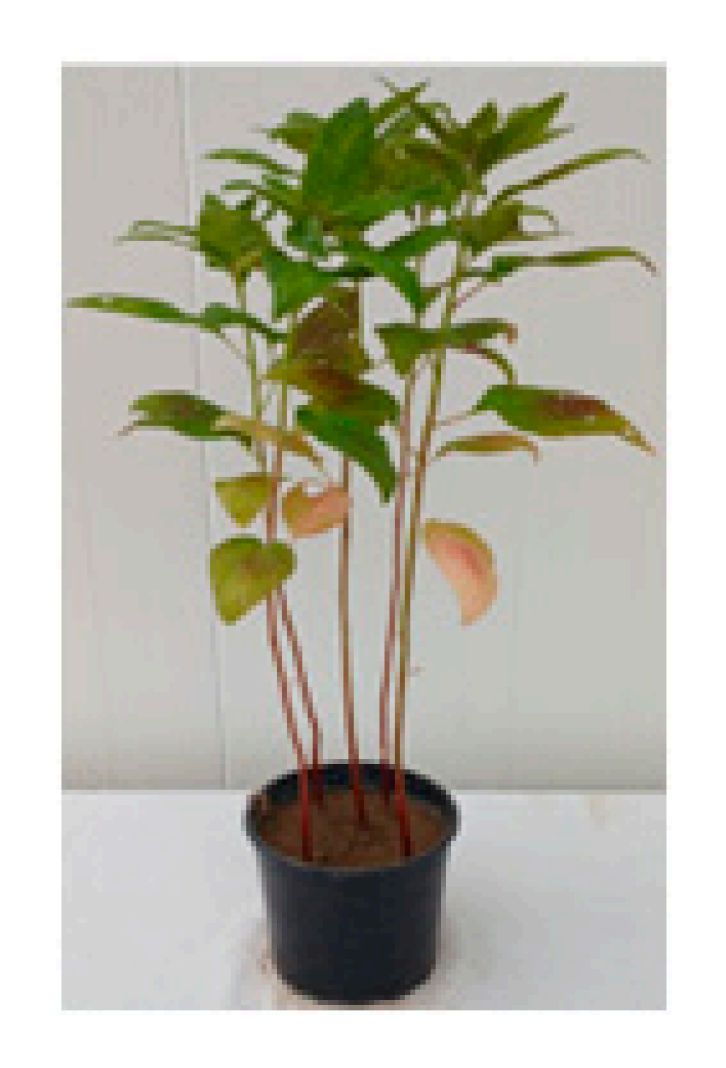	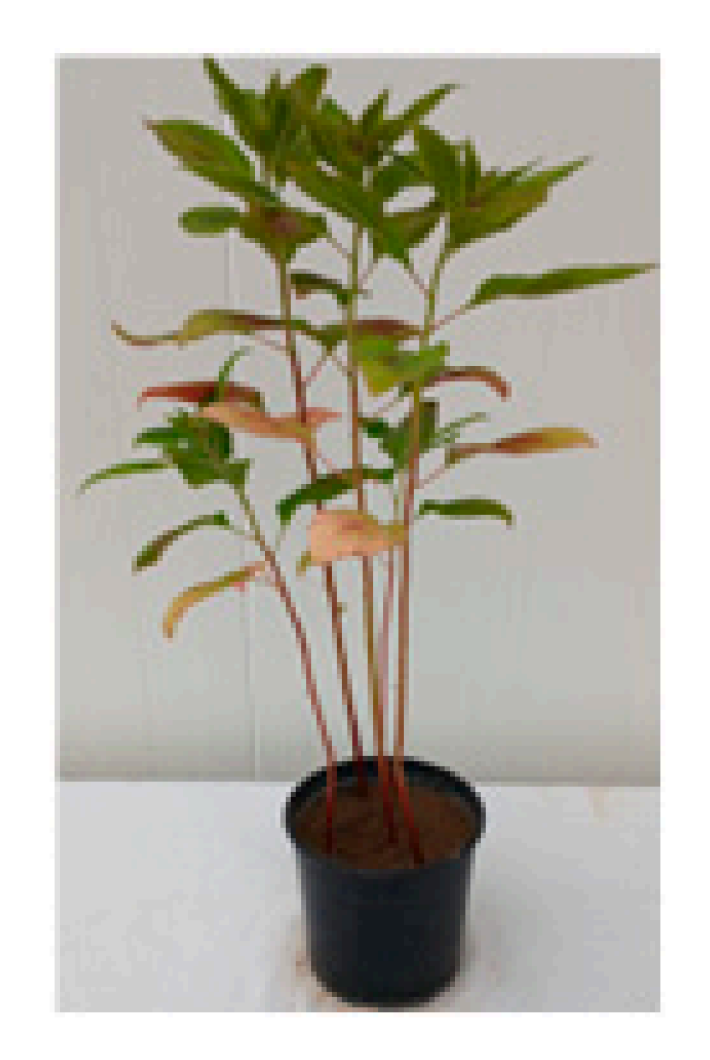	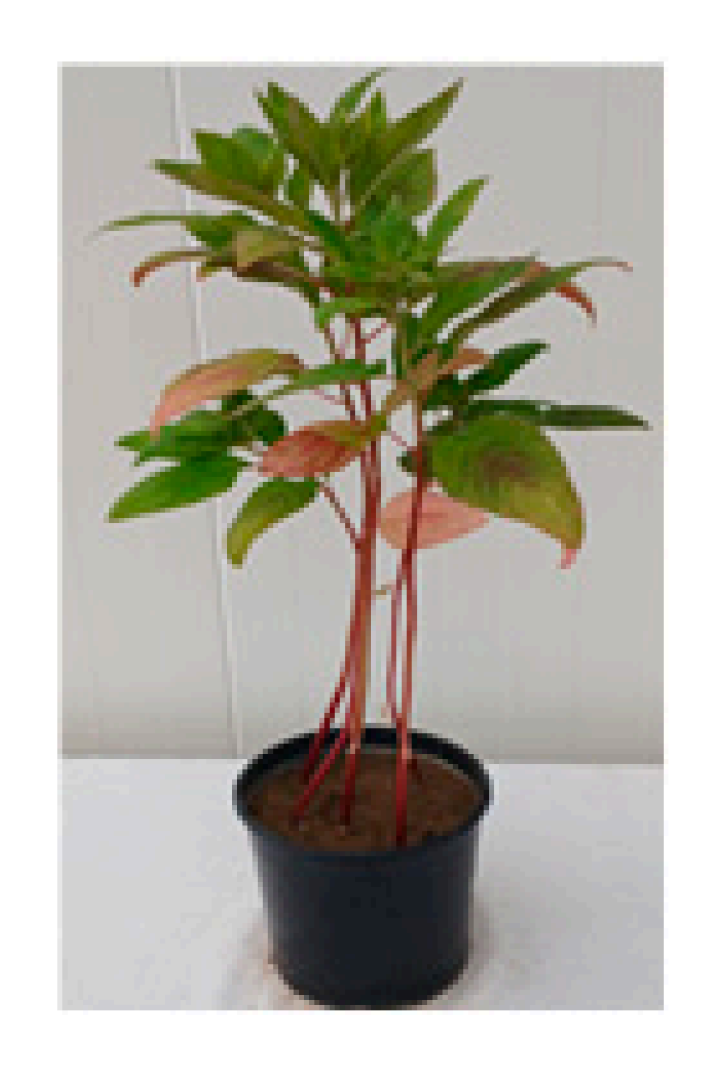	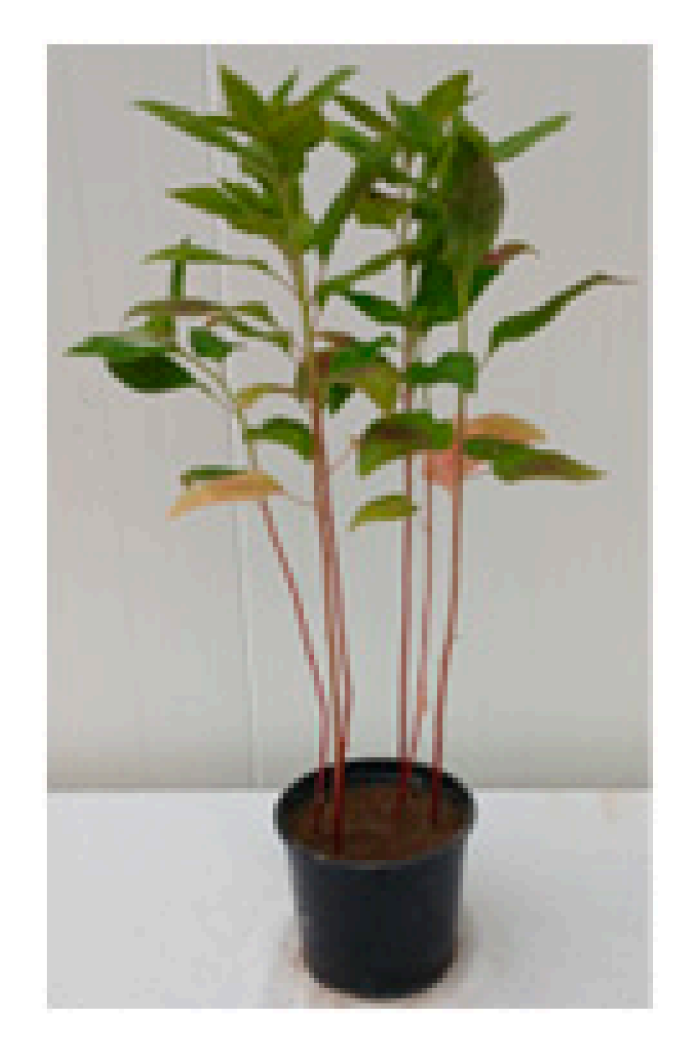
SL	71.115 ± 4.095 ^a^	55.110 ± 4.467 ^b^	59.915 ± 6.856 ^a^	59.840 ± 1.658 ^a^	59.600 ± 3.308 ^a^	56.460 ± 2.202 ^b^
FBAP	68.948 ± 3.450 ^c^	72.180 ± 9.967 ^b^	80.650 ± 8.294 ^a^	60.805 ± 1.917 ^d^	72.193 ± 8.952 ^b^	66.175 ± 4.764 ^c^

SL—stem length (cm); FBAP—fresh biomass of aboveground parts (g pot^−1^). Homogeneous groups were calculated separately for each parameter. C—soil without the addition of perfluorotetradecanoic acid and biostimulants; PA—soil supplemented with perfluorotetradecanoic acid; C_Sh—soil supplemented with the Shigeki biostimulant; PA_Sh—soil supplemented with perfluorotetradecanoic acid and the Shigeki biostimulant; C_Am—soil supplemented with the Aminoprim biostimulant; PA_Am—soil supplemented with perfluorotetradecanoic acid and the Aminoprim biostimulant. Homogeneous groups designated by letters (a–d) were calculated separately for each parameter.

**Table 2:** Fresh biomass root parts (FBRP) of *Amaranthus cruentus* (g pot^−1^) at the BBCH 39 growth stage.

Parameter	Treatments					
	C	PA	C_Sh	PA_Sh	C_Am	PA_Am
	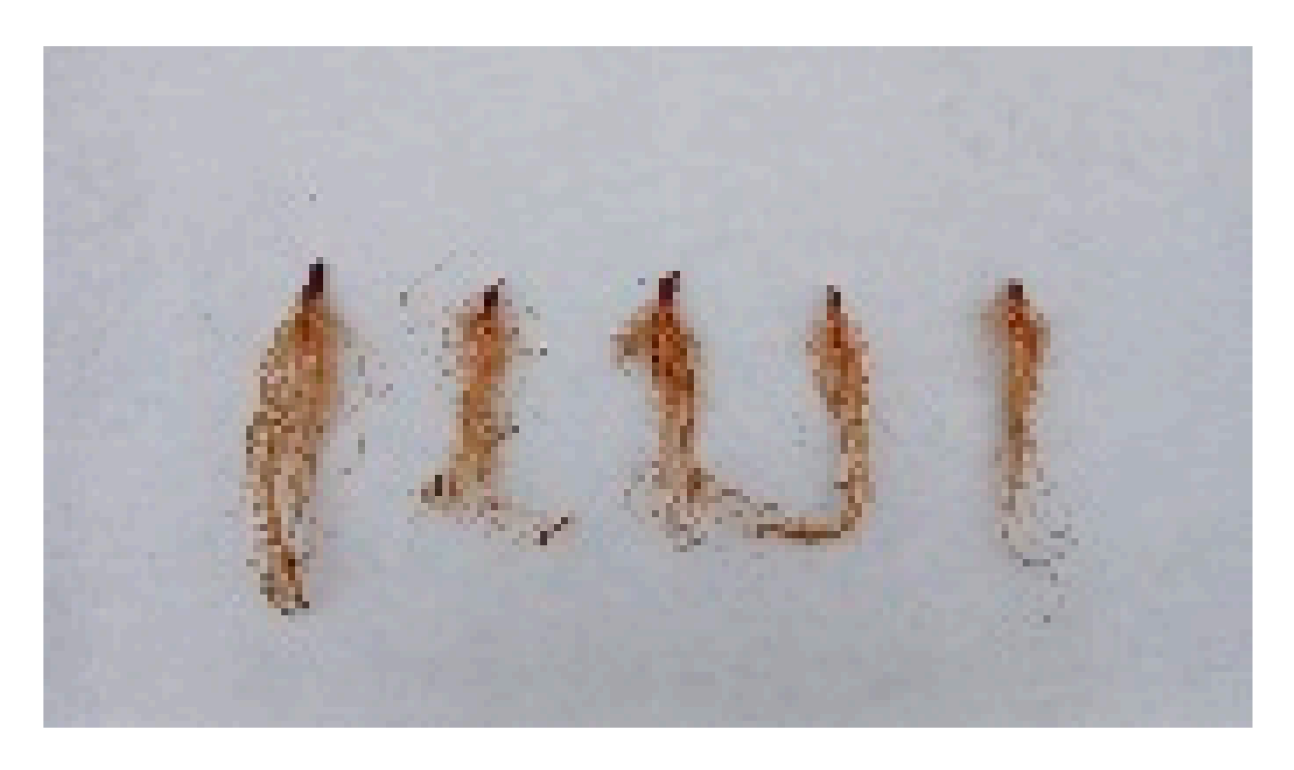	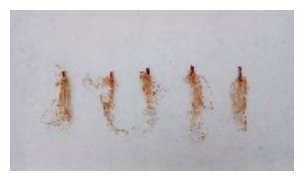	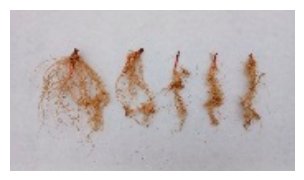	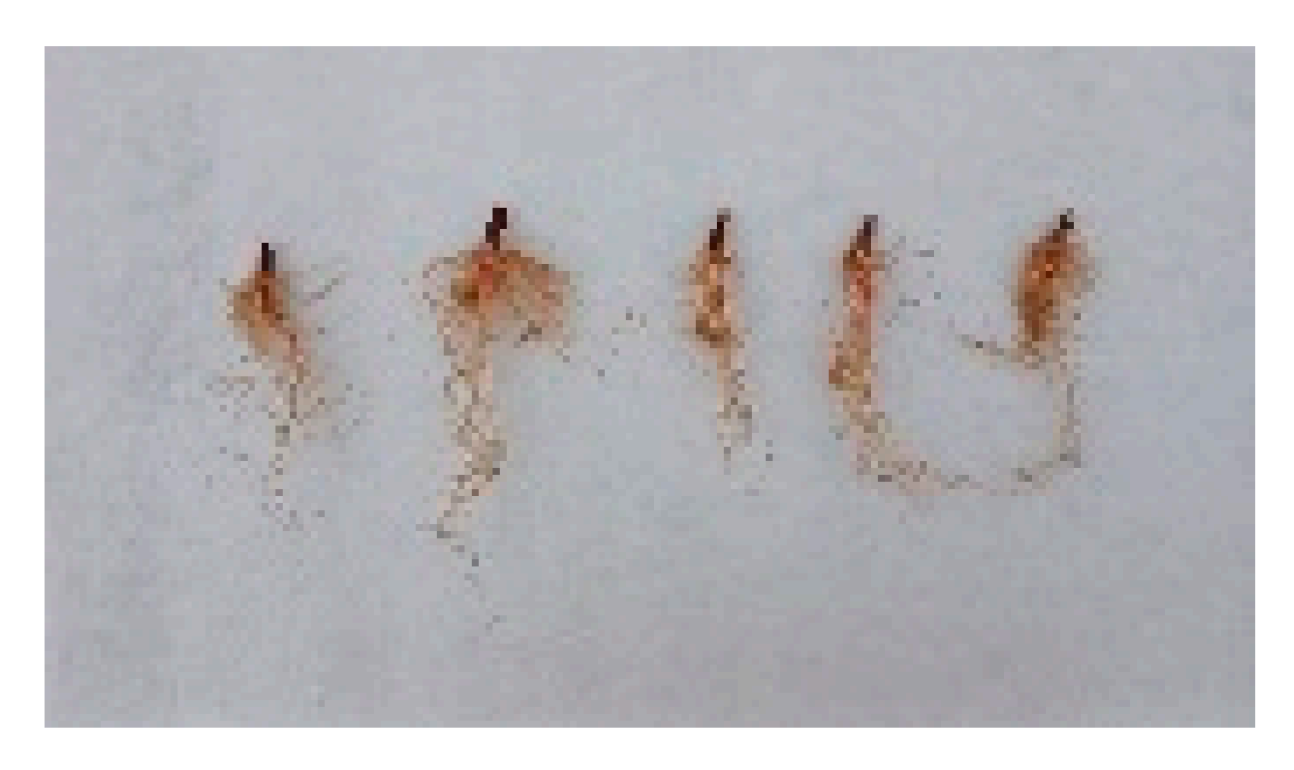	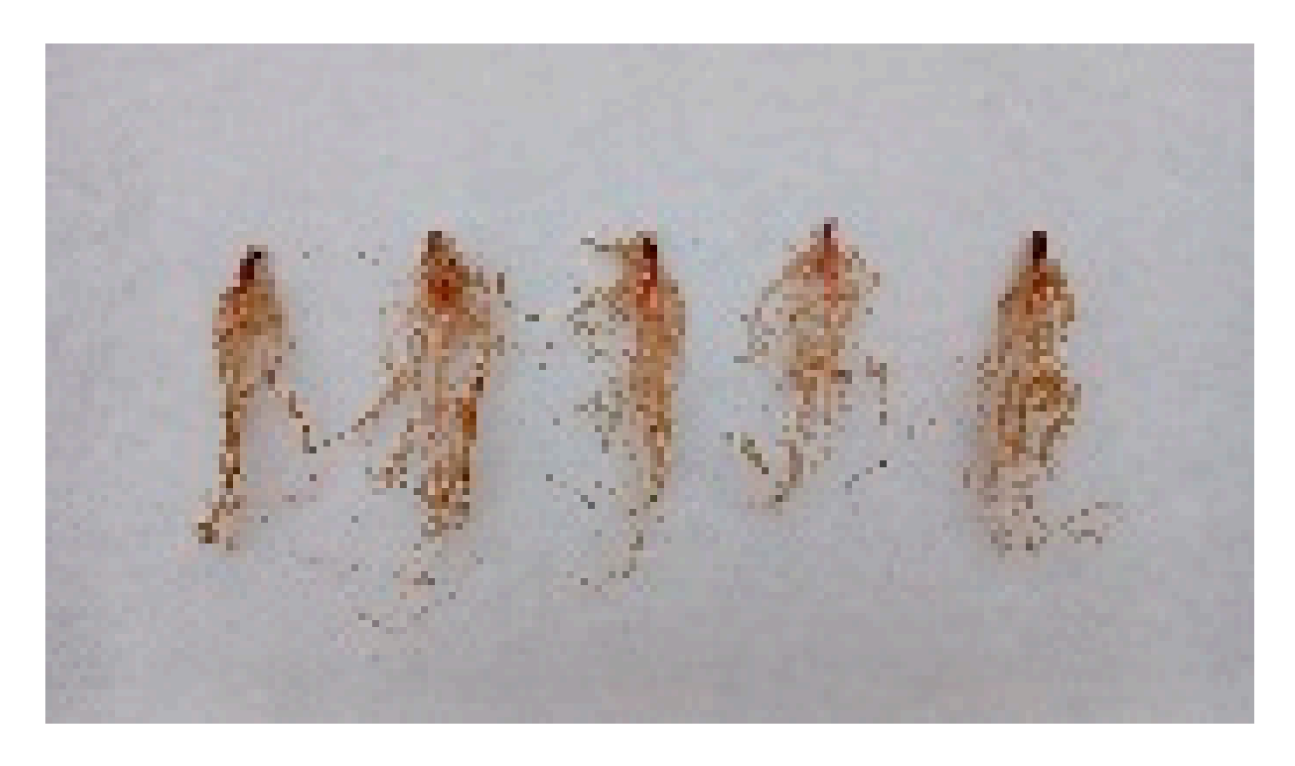	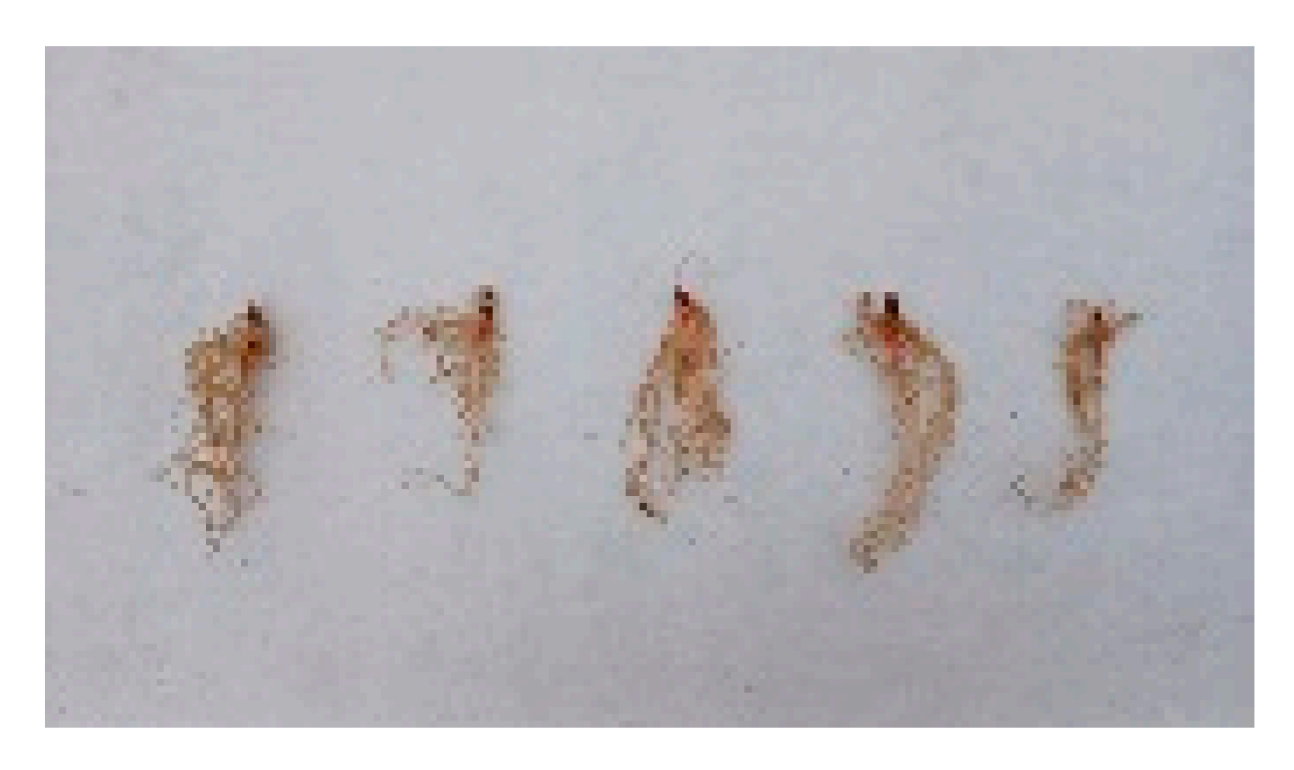
FBRP	22.418 ± 5.508 ^b^	21.330 ± 2.694 ^b^	25.810 ± 3.328 ^a^	19.383 ± 1.165 ^c^	20.875 ± 1.954 ^c^	20.963 ± 0.624 ^c^

Abbreviations are explained in Table 1. Homogeneous groups were designated by letters (a–c).

**Table 3:** Abbreviations used for the experimental treatments.

Abbreviations for Experimental Treatments	Abbreviations Used for Experimental Treatments
C	soil without the addition of perfluorotetradecanoic acid and biostimulants
PA	soil supplemented with perfluorotetradecanoic acid
C_Sh	soil supplemented with the Shigeki biostimulant
PA_Sh	soil supplemented with perfluorotetradecanoic acid and the Shigeki biostimulant
C_Am	soil supplemented with the Aminoprim biostimulant
PA_Am	soil supplemented with perfluorotetradecanoic acid and the Aminoprim biostimulant

**Table 4:** Physicochemical properties of the model soil.

Type Soil	Granulometric Composition (%)			pH	HAC	EBC	CEC	BS	C_org_	N_total_
	Sand	Silt	Clay		mmol^+^ kg^−1^				g kg^−1^	
sl	62.47	37.49	0.04	4.73	15.19	28.00	43.19	64.83	7.05	1.05

sl—loamy sand; sand—particle size range from 2000 to 50 μm; silt—particle size range from 50 to 2 μm; clay—particle size < 2 μm; HAC—hydrolytic acidity; EBC—exchangeable base cations; CEC—cation exchange capacity; BS—base saturation; C_org_—total organic carbon; N_total_—total nitrogen.

**Table 5:** PCR primers used for fungal DNA amplification.

Species Type	Region	Amplified Region	Primer	
			Forward	Reverse
Fungi	ITS	ITS1-1F	CTTGGTCATTTAGAGGAAGTAA	GCTGCGTTCTTCATCGATGC
		ITS1-5F	GGAAGTAAAAGTCGTAACAAGG	GCTGCGTTCTTCATCGATGC
		ITS2	GCATCGATGAAGAACGCAGC	TCCTCCGCTTATTGATATGC

## Data Availability

The original contributions presented in this study are included in the article.

## Supplementary Materials

The following supporting information can be downloaded at https://www.mdpi.com/article/10.3390/molecules31193385/s1, Table S1: The number of reads assigned to individual fungal trophic modes and the corresponding *p*-values for the analyzed associations; Table S2: Quality assessment of fungal sequence reads.

## Abbreviations

The following abbreviations are used in this manuscript:

PFASs	Per- and polyfluoroalkyl substances
C	Soil without the addition of perfluorotetradecanoic acid and biostimulants
PA	Soil supplemented with perfluorotetradecanoic acid
C_Sh	Soil supplemented with the Shigeki biostimulant
PA_Sh	Soil supplemented with perfluorotetradecanoic acid and the Shigeki biostimulant
C_Am	Soil supplemented with the Aminoprim biostimulant
PA_Am	Soil supplemented with perfluorotetradecanoic acid and the Aminoprim biostimulant
