# Supplementary material for: Biostimulant Modulation of Soil Mycobiome Structure and Diversity and Amaranthus cruentus Growth Under Perfluorotetradecanoic Acid Stress
Source: Molecules. 2026 Sep 23;31(19):3385. doi: 10.3390/molecules31193385 (PMC13666479; doi:10.3390/molecules31193385)
Supplement: Supplementary file 1 [file molecules-31-03385-s001.zip › molecules-4482511-supplementary.pdf]

molecules

MDPI

Supplementary Material

Biostimulant Modulation of Soil Mycobiome Structure and Diversity and *Amaranthus cruentus* Growth under Perfluorotetradecanoic Acid Stress

Małgorzata Baćmaga [1], Jadwiga Wyszkowska [1,*], Edyta Boros-Lajszner [1] and Jan Kucharski [1]

[1] Department of Soil Science and Microbiology, Faculty of Agriculture and Forestry, University of Warmia and Mazury in Olsztyn, Plac Łódzki 3, 10-727 Olsztyn, Poland; m.bacmaga@uwm.edu.pl (M.B.); edyta.boros@uwm.edu.pl (E.B.-L.); jan.kucharski@uwm.edu.pl (J.K.)
* Correspondence: jadwiga.wyszkowska@uwm.edu.pl

Table S1. The number of reads assigned to individual fungal trophic modes and the corresponding *p*-values for the analyzed associations.

Functional prediction	C	PA	C_Sh	PA_Sh	C_Am	PA_Am	*p*-value
Unassigned	6679	4884	6608	22970	13917	13057	0.120
Undefined_Saprotroph	16876	21504	19249	2359	9129	8079	0.539
Plant_Pathogen-Soil_Saprotroph-Wood_Saprotroph	1026	619	599	885	1162	2555	0.027
Undefined_Saprotroph-Wood_Saprotroph	254	487	205	1098	1005	3013	0.657
Dung_Saprotroph-Undefined_Saprotroph-Wood_Saprotroph	1722	663	971	868	2005	702	1.000
Plant_Pathogen	74	24	116	37	116	63	0.020
Ericoid_Mycorrhizal	305	49	185	94	341	150	0.265
Arbuscular_Mycorrhizal	0	0	1	2	2	4	0.011
Animal_Pathogen-Endophyte-Fungal_Parasite-Plant_Pathogen-Wood_Saprotroph	654	17	43	58	281	28	0.913
Fungal_Parasite	575	83	242	29	248	133	0.334
Fungal_Parasite-Undefined_Saprotroph	184	119	193	46	148	274	0.485
Ectomycorrhizal	49	28	49	15	7	40	0.005
Dung_Saprotroph	131	49	63	27	88	237	0.029
Animal_Pathogen-Soil_Saprotroph	3	0	3	3	21	76	0.500
Animal_Pathogen-Dung_Saprotroph-Endophyte-Plant_Saprotroph-Soil_Saprotroph-Wood_Saprotroph	7	11	0	45	65	17	0.110
Endophyte-Plant_Pathogen	2	13	4	4	5	42	0.166
Endophyte	23	5	20	10	14	28	0.328
Plant_Pathogen-Undefined_Saprotroph	0	0	0	2	7	12	0.189
Animal_Pathogen	8	17	21	16	22	27	0.033
Dung_Saprotroph-Endophyte-Litter_Saprotroph-Undefined_Saprotroph	0	0	0	7	0	21	0.235

 https://doi.org/10.3390/molecules31193385

Animal_Pathogen-Dung_Saprotroph-Endophyte-Plant_Pathogen-Undefined_Saprotroph	8	6	6	7	0	5	0.923
Leaf_Saprotroph	0	0	0	3	1	16	0.110
Animal_Pathogen-Endophyte-Plant_Saprotroph-Soil_Saprotroph	0	6	3	1	0	0	0.018
Animal_Pathogen-Plant_Pathogen-Soil_Saprotroph-Undefined_Saprotroph	8	0	2	2	2	0	0.917
Animal_Pathogen-Clavicipitaceous_Endophyte-Fungal_Parasite	0	2	6	0	2	0	0.141
Plant_pathogenic_on_polen	0	0	0	0	0	5	0.363
Wood_Saprotroph	0	0	0	3	0	2	0.185
Soil_Saprotroph	3	3	0	0	3	2	0.028
Plant_Pathogen-Wood_Saprotroph	0	2	0	0	0	0	0.788
Endophyte-Litter_Saprotroph-Wood_Saprotroph	0	0	2	0	0	0	0.363
Animal_Endosymbiont-Undefined_Saprotroph	0	0	0	0	0	2	0.363

C—soil without the addition of perfluorotetradecanoic acid and biostimulants; PA—soil supplemented with perfluorotetradecanoic acid; C_Sh—soil supplemented with the Shigeki biostimulant; PA_Sh—soil supplemented with perfluorotetradecanoic acid and the Shigeki biostimulant; C_Am—soil supplemented with the Aminoprim biostimulant; PA_Am—soil supplemented with perfluorotetradecanoic acid and the Aminoprim biostimulant.

Table S2. Quality assessment of fungal sequence reads.

Object	RawPE	Combined	Qualified	Nochime	Base(nt)	Avglen(nt)	GC (%)	Q20 (%)	Q30 (%)
C	41,606	41,422	41,365	30,707	7,537,747	245.47	56.47	99.54	97.88
PA	66,266	66,032	65,957	56,216	13,614,435	242.18	53.63	99.59	98.14
C_Sh	66,176	65,865	65,753	53,220	13,109,892	246.33	57.20	99.51	97.73
PA_Sh	69,971	69,129	68,629	62,490	17,380,101	278.13	38.86	98.99	95.89
C_Am	66,538	65,868	65,740	49,506	12,618,779	254.89	49.55	99.50	97.68
PA_Am	64,367	63,840	63,753	45,061	10,877,444	241.39	53.12	99.58	98.08

RawPE—raw paired-end reads before filtering; Combined—reads after paired-end merging; Qualified—quality-filtered reads meeting the predefined criteria; Nochime—reads remaining after chimera removal; Base (nt)—total number of nucleotides; AvgLen (nt)—mean sequence length; GC (%)—percentage of G and C bases; Q20 (%)—percentage of bases with a Phred quality score ≥20; Q30 (%)—percentage of bases with a Phred quality score ≥30. C—soil without the addition of perfluorotetradecanoic acid and biostimulants; PA—soil supplemented with perfluorotetradecanoic acid; C_Sh—soil supplemented with the Shigeki biostimulant; PA_Sh—soil supplemented with perfluorotetradecanoic acid and the Shigeki biostimulant; C_Am—soil supplemented with the Aminoprim biostimulant; PA_Am—soil supplemented with perfluorotetradecanoic acid and the Aminoprim biostimulant.

https://doi.org/10.3390/xxxxx
